# Antimicrobial Activity of Naphthyridine Derivatives

**DOI:** 10.3390/ph17121705

**Published:** 2024-12-17

**Authors:** Anna Wójcicka, Marcin Mączyński

**Affiliations:** Department of Organic Chemistry and Pharmaceutical Technology, Faculty of Pharmacy, Wroclaw Medical University, 211A Borowska Str., 50-556 Wroclaw, Poland; marcin.maczynski@umw.edu.pl

**Keywords:** naphthyridine derivatives, structure–activity relationships, antimicrobial activity, nalidixic acid

## Abstract

To combat the problem of the increasing drug resistance of microorganisms, it is necessary to constantly search for new medicinal substances that will demonstrate more effective mechanisms of action with a limited number of side effects. Naphthyridines are N-heterocyclic compounds containing a fused system of two pyridine rings, occurring in the form of six structural isomers with different positions of nitrogen atoms, which exhibit a wide spectrum of pharmacological activity, in particular antimicrobial properties. This review presents most of the literature data about the synthetic and natural naphthyridine derivatives that have been reported to possess antimicrobial activity.

## 1. Introduction

**Figure 1:**
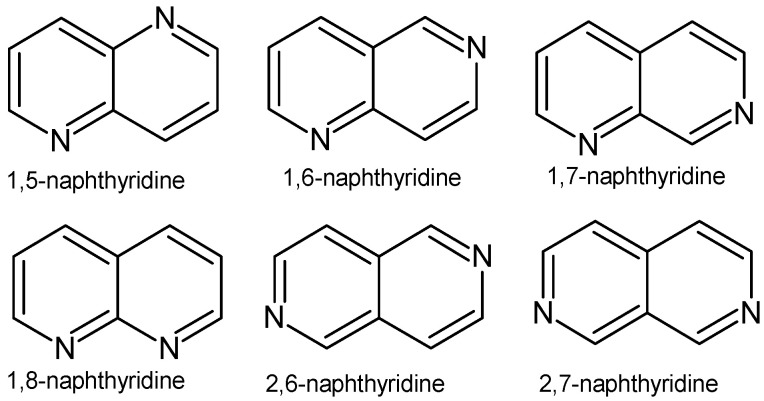
Naphthyridine isomers.

Infectious diseases are an increasing problem in modern medicine. The growing drug resistance of bacterial and pathogenic fungi strains is one of the greatest threats to human health and life. Antimicrobial resistance may appear within a few years after the introduction of a new therapeutic agent to treatment. In order to combat growing drug resistance and increase the effectiveness and safety of therapy, it is necessary to constantly search for new medicinal substances that demonstrate more effective mechanisms of action with a limited number of adverse effects. Researchers are investigating new antimicrobial compounds using traditional techniques such as broth dilution, well diffusion, and disc diffusion, and as well as various antimicrobial assays, such as cross-streaking, poisoned food, time–kill kinetics, co-cultures, and resazurin assays. More advanced techniques such as impedance analysis, flow cytometry, and bioluminescence techniques provide rapid and accurate results and can show the effect of antimicrobials on cellular integrity [1]. Drugs can be obtained by isolating the desired compounds from natural products, developing new methods of organic synthesis, or modifying existing chemical structures. Naphthyridines are N-heterocyclic compounds, which are also called “diazanaphthalenes” or “benzodiazines”, due to the presence of a fused system of two pyridine rings, occurring in the form of six isomers with different positions of nitrogen atoms (Figure 1).

**Figure 2:**
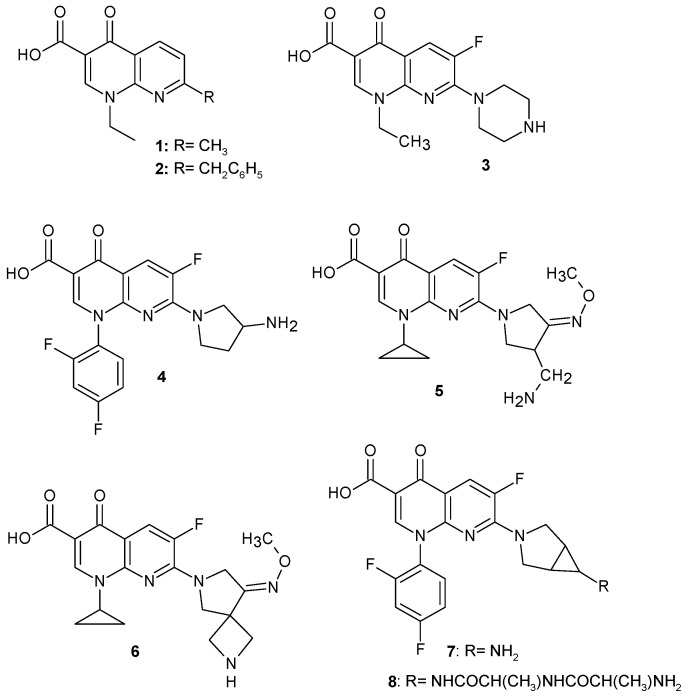
Nalidixic acid and its derivatives used in medicine.

According to a previous report, naphthyridine derivatives exhibit a broad spectrum of pharmacological activity, including anti-infective, anti-cancer, neurological, cardiovascular, and immunological effects [2]. Their wide range of effects makes them a promising subject of scientific research for their therapeutic use. The first naphthyridine derivative introduced into clinical practice in 1967 as an antibacterial agent was nalidixic acid **1** (Figure 2). This review presents naphthyridine derivatives with antimicrobial activity.

## 2. 1,8-Naphthyridine Derivatives

### 2.1. Antimicrobial Activity of Nalidixic Acid Derivatives

4-Oxo-1,8-naphthyridine-3-carboxylic acid is the basic structure of several commercially available antibacterial drugs. The first drug from this group of compounds was obtained and reported by G. Lesher et al. [3]—1-ethyl-7-methyl-4-oxo-1,8-naphthyridine-3-carboxylic acid **1**, called nalidixic acid (Figure 2). This compound was introduced into medicine in 1967 for the treatment of urinary tract infections caused by Gram-negative bacteria. Nalidixic acid **1** selectively and reversibly blocks DNA replication in bacteria by inhibiting the A subunit of bacterial DNA gyrase [4]. The marketing authorization for nalidixic acid has been suspended in the EU and other countries due to side effects. However, in the following years, as a result of structural modification, many nalidixic acid derivatives with antibacterial activity were obtained, and some of them were introduced into medicine (Figure 2). 

Amfonelic acid **2** is 7-benzyl derivative of nalidixic acid, but despite its antibiotic properties, it is used as a dopaminergic stimulant [5].

In the search for new structural analogues of fluoroquinolone antibiotics, a fluorine atom was introduced into the 1,8-naphthyridine ring, and a number of 6-fluoro-4-oxo-1,8-naphthyridine-3-carboxylic acid derivatives with antimicrobial activity were obtained, including several medicinal substances, **3**–**8** (Figure 2).

Another analogue containing a piperazine ring was enoxacin: 1-ethyl-6-fluoro-4-oxo-7-(piperazin-1-yl)-1,4-dihydro-1,8-naphthyridine-3-carboxylic acid **3**, whose mechanism of action is to block bacterial DNA replication by binding to DNA gyrase and which inhibits microRNA expression in many Gram-positive and Gram-negative bacteria [6]. Enoxacin **3** is a structural analogue of norfloxacin (an antibiotic from the fluoroquinolone group) and can be used to treat a wide range of infections, in particular gastroenteritis, respiratory tract infections, urinary tract infections, and gonorrhoea under the brand name Penetrex*@* [7]. 

The introduction of a pyrrolidine ring into the structure resulted in obtaining a number of compounds with antibacterial activity. Some of them, **4**–**8** (Figure 2), were introduced in medicine.

Tosufloxacin: 7-(3-aminopyrrolidin-1-yl)-1-(2,4-difluorophenyl)-6-fluoro-4-oxo-1,4-dihydro-1,8-naphthyridine-3-carboxylic acid **4** was developed by Toyama Chemical and introduced to the market in Japan under the trade name Ozex@. The use of this drug has been limited due to side effects: severe thrombocytopenia, nephritis, and hepatotoxicity [8]. 

Gemifloxacin mesylate: (*R*,*S*)-7-(3-aminomethyl-4-*syn*-methoxyimino-1-pyrrolidinyl)-1-cyclopropyl-6-fluoro-1,4-dihydro-4-oxo-1,8-naphthyridine-3-carboxylic acid **5** is indicated for the treatment of acute bacterial exacerbation of chronic bronchitis and mild to moderate community-acquired pneumonia caused by *Staphylococcus aureus*, *Staphylococcus pyogenes*, *Haemophilus influenza*, *Klebsiella pneumoniae*, *Legionella pneumophila*, *Moraxella catarrhalis*, *Chlamydia pneumoniae*, and *Mycoplasma pneumoniae*, as well as the multi-drug resistant strain *S. pneumoniae*, under the brand name Factive@ [9,10]. Gemifloxacin acts by inhibiting DNA replication by binding itself to DNA gyrase and topoisomerase IV [11]. 

Zabofloxacin: 1-cyclopropyl-6-fluoro-1,4-dihydro-7-[8-(methoxyimino)-2,6-diazaspiro [3.4]oct-6-yl]-4-oxo-1,8-naphthyridine-3-carboxylic acid **6** is an investigational antibiotic for treating multidrug-resistant infections caused by Gram-positive bacteria and *Neisseria gonorrhoeae* [12,13,14,15].

Trovafloxacin: 7-[(1*S*,5*R*)-6-amino-3-azabicyclo[3.1.0]hexan-3-yl]-1-(2,4-difluorophenyl)-6-fluoro-4-oxo-1,8-naphthyridine-3-carboxylic acid **7** is an inhibitor of topoisomerase IV and DNA synthesis. This drug has been marketed under the trade name Trovan@ by Pfizer since 1997 as a broad-spectrum antibacterial drug used against Gram-positive streptococci and Gram-negative pathogens [16], including a resistant strain of *Neisseria gonorrhoeae* [17].

Alatrofloxacin is the alaninamide of trovofloxacin: 7-[(1*R*,5*S*)-6-{[(2*S*)-1-{[(2*S*)-2-aminopropanoyl]amino}-1-oxopropan-2-yl]amino}-3-azabicyclo[3.1.0]hexan-3-yl]-1-(2,4-difluorophenyl)-6-fluoro-4-oxo-1,8-naphthyridine-3-carboxylic acid **8**, which was also developed by Pfizer and marketed under the brand name Trovan@ IV as a prodrug quickly hydrolyzed to trovofloxacin. Trovafloxacin **7** and alatrofloxacin **8** were both withdrawn from the market due to the risk of hepatotoxicity [18]. 

**Figure 3:**
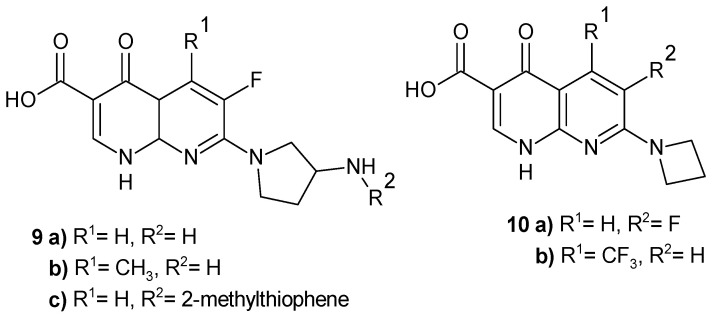
Nalidixic acid derivatives **9**–**10**.

In the Abbott laboratory [19], new nalidixic acid derivatives with pyrrolidine and azetidine moieties were obtained, which turned out to be novel ribosome inhibitors (NRIs) that selectively inhibit bacterial protein synthesis by structurally disrupting the tRNA/30S complex at the decoding site [20]. Pyrrolidine derivatives **9a** and **9c** (Figure 3) exhibit selective and broad-spectrum antibacterial activity, including drug-resistant respiratory pathogens, and are nontoxic to human cell lines [21]. Among the fluorinated 1,8-naphthyridines with an azetidine moiety, the most potent compounds were derivatives **10a** and **10b** (Figure 3) [20]. 

**Figure 4:**
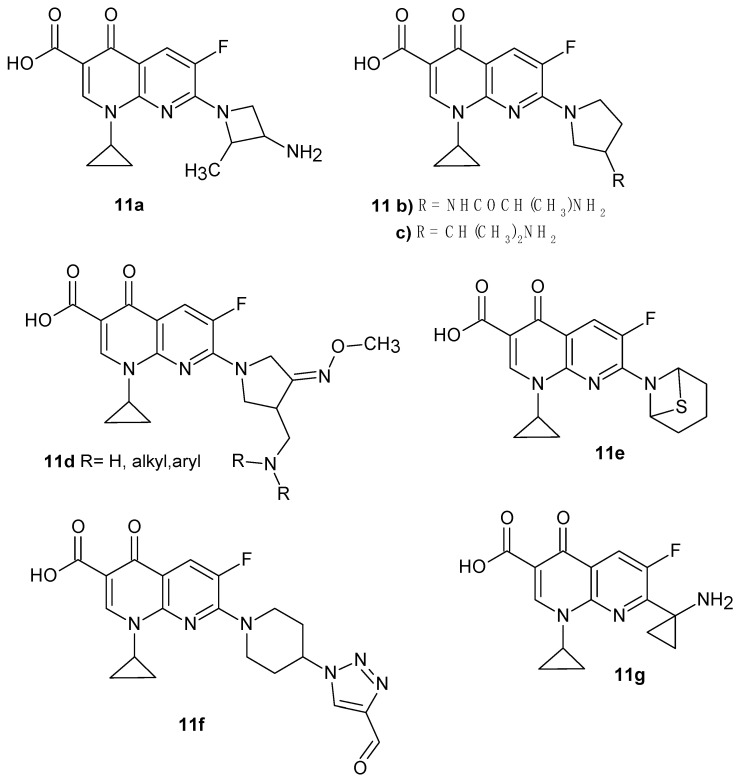
1-Cyclopropyl-6-fluoro-1,4-dihydro-4-oxo-1,8-naphthyridine-3-carboxylic acid derivatives.

By introducing a cyclopropyl substituent into the structure of fluorinated 1,8-naphthyridine derivatives, some active compounds were obtained (Figure 4). The highest antimicrobial activity was shown by compounds **11a**–**c**. 7-[3-(*R*)-amino-2-(*S*)-methyl-1-azetidinyl]-1-cyclopropyl-1,4-dihydro-6-fluoro-4-oxo-1,8-naphthyridine-3-carboxylic acid **11a**, synthesized at Laboratorios Dr. Esteve S.A., Barcelona, Spain [22], turned out to be more active than the comparative drugs ciprofloxacin, fleroxacin, lomefloxacin, norfloxacin, enoxacin, and ofloxacin against *Staphylococcus*, *Actinetobacter*, *Pseudomonas aeruginosa*, *Xanthomonas maltophilia*, *Neisseria gonorrhoeae*, and *Enterococcus*, including also some ciprofloxacin-resistant strains [23]. 7-(3-Amino-1-pyrrolidinyl)-1-cyclopropyl-6-fluoro-1,4-dihydro-4-oxo-1,8-naphthyridine-3-carboxylic acid **11b** was developed at the Parke-Davis Pharmaceutical Research Division of Warner-Lambert Co. [24] and used in clinical trials [25,26]. Derivative **11b** is active against various resistant bacteria, comparable to the activity of ciprofloxacin or ofloxacin against *E. coli*, but much more potent against *Staphylococci* [27]. Clinical trials showed that compound **11b** and its analogue 7-[(3*R*)-3-(2-aminopropan-2-yl)pyrrolidin-1-yl]-1-cyclopropyl-6-fluoro-4-oxo-1,4-dihydro-1,8-naphthyridine-3-carboxylic acid **11c** showed high activity against antibiotic-resistant *Enterococcus* strains [26].

Hong et al. [28] synthesized new pyrrolidine derivatives of nalidixic acid **11d** (Figure 4), which showed very strong antibacterial activity against Gram-negative and Gram-positive bacteria, including methicillin-resistant *S. aureus*. Obtained by the same scientist, (*R*,*S*)-7-(3-aminomethyl-4-*syn*-methoxyimino-1-pyrrolidinyl)-1-cyclopropyl-6-fluoro-1,4-dihydro-4-oxo-1,8-naphthyridine-3-carboxylic acid **5** (Figure 2) was introduced into medicine as gemifloxacin.

7-(2-Thia-5-azabicyclo[2.2.1]heptan-5-yl)-1-cyclopropyl-6-fluoro-4-oxo-1,4-dihydro-1,8-naphthyridine-3-carboxylic acid **11e**, obtained by X. Huang et al. [29], showed very good antibacterial activity against multidrug-resistant strains of *Streptococcus pneumonia* in comparison with ciprofloxacin and vancomycin. 1-Cyclopropyl-6-fluoro-7-(4-(4-formyl-1*H*-1,2,3-triazol-1-yl)piperidin-1-yl)-4-oxo-1,4-dihydro-1,8-naphthyridine-3-carboxylic acid **11f** exhibited a comparable activity against multidrug-resistant strains, especially against *S. aureus* and *Staphylococcus epidermidis*, compared to ciprofloxacin and vancomycin [30].

**Figure 5:**
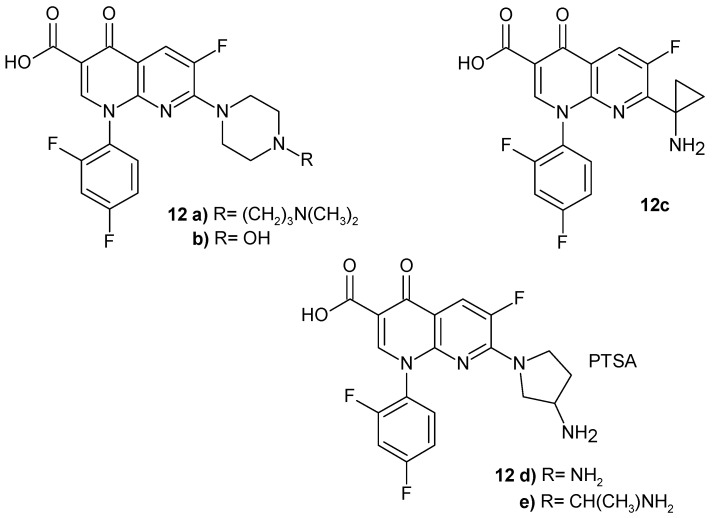
1-(2,4-Difluorophenyl)-6-fluoro-4-oxo-1,8-naphthyridine-3-carboxylic acid derivatives **12a**–**e**.

Y. Todo et al. [31] synthesized 7-(1-aminocyclopropyl)-1-cyclopropyl-6-fluoro-4-oxo-1,4-dihydro-1,8- naphthyridine-3-carboxylic acid **11g** (Figure 4) and 7-(1-aminocyclopropyl)-1-(2,4-difluorophenyl)-6-fluoro-4-oxo-1,4-dihydro-1,8-naphthyridine-3-carboxylic acid **12c** (Figure 5), which exhibited a comparable antibacterial activity against Gram-positive and Gram-negative bacteria strains compared to ciprofloxacin and ofloxacin.

The study results showed that the combination of a cyclopropyl group or a substituted phenyl group at N-1 and a 3-amino-2-methyl-1-azetidinyl group at C-7 provided the best overall antibacterial, pharmacokinetic, and physicochemical properties of the naphthyridine derivatives tested [32].

H.K. Gencer et al. [33] synthesized new derivatives of 1-(2,4-difluorophenyl)-6-fluoro-4-oxo-1,8-naphthyridine-3-carboxylic acid **12a**–**e** (Figure 5) with antibacterial activity. Compound **12a** with a piperazine moiety is a strong inhibitor of DNA gyrase and is non-genotoxic and less cytotoxic compared to the reference drugs: trovafloxacin, moxifloxacin, and ciprofloxacin. 

J. Domagala et al. [34] also synthesized new analogues of 2,4-difluorophenyl derivatives **12**, the most active of which was 7-[3-(1-aminoethyl)pyrrolidin-1-yl]-1-(2,4-difluorophenyl)-6-fluoro-4-oxo-1,4-dihydro-1,8-naphthyridine-3-carboxylic acid **12e** (Figure 5), demonstrating the best overall combination of safety and effectiveness.

(T-3262) p-toluenesulfonic acid salt of DL-7-(3-amino-1-pyrrolidinyl)-1-(2,4-difluorophenyl)-6-fluoro-1,4-dihydro-4-oxo-1,8-naphthyridine-3-carboxylic acid monohydrate **12d** (Figure 5) showed excellent activity against Gram-positive bacteria strains, particularly *Streptococci* [35]. It was found to be comparable to ciprofloxacin and ofloxacin [36]. 

Cooper et al. [37] synthesized a series of 1-(2,4-difluorophenyl)-6-fluoro-4-oxo-1,8-naphthyridine-3-carboxylic acid derivatives with a piperazine moiety that displayed in vivo activity against *S. aureus.* The most active was compound **12b** (Figure 5).

**Figure 6:**
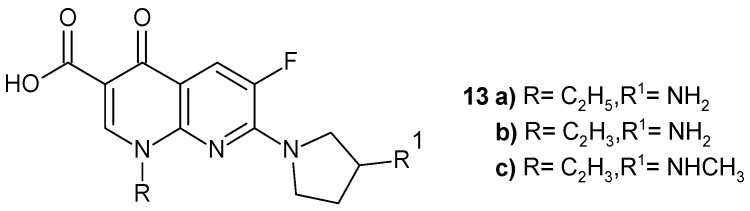
Structures of derivatives **13a**–**c**.

Synthesized by Egawa et al. [38] 1-alkyl-7-(3-amino-1-pyrrolidinyl)-6-fluoro-1,4-dihydro-4-oxo-1,8-naphthyridine-3-carboxylic acids **13a**–**b** and 1-vinyl-7-[3-(methylamino)-1-pyrrolidinyl] analogue **13c** (Figure 6) were found to be more active than enoxacin. 

**Figure 7:**
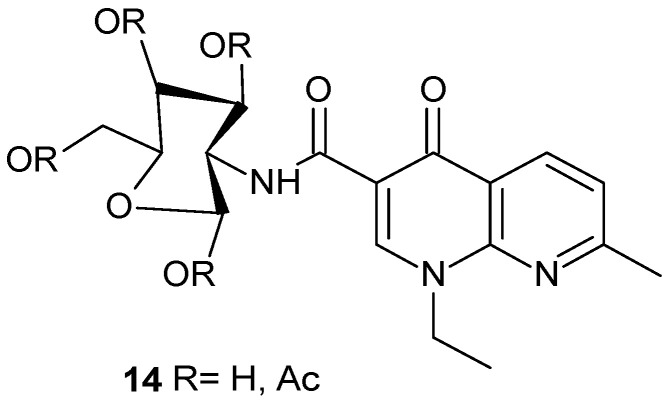
Nalidixic acid-D-(+)-glucosamine conjugate **14**.

Biological studies aimed at assessing the antimicrobial activity of nalidixic acid-D-(+)-glucosamine conjugate **14** (Figure 7) indicated an improvement in the activity of the synthesized derivatives against various strains of resistant bacteria, while demonstrating a lower cytotoxic effect [39].

**Figure 8:**
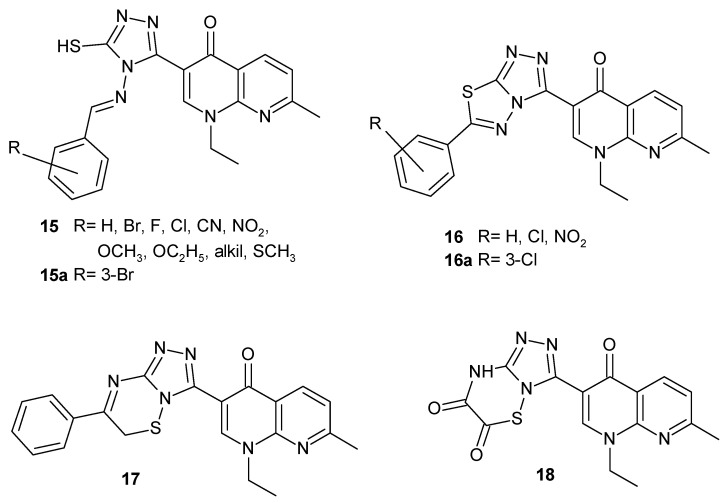
Nalidixic acid-based 1,2,4-triazole derivatives **15**–**18**.

N. Aggarwal et al. [40] synthesized new nalidixic acid derivatives with 1,2,4-triazole moiety **15**–**18** (Figure 8). Many of these compounds possessed good antibacterial and slightly less antifungal activity, but the most potent were 3-{4-(3-bromobenzylideneamino)-5-mercapto-4*H*-1,2,4-triazol-3-yl}-1-ethyl-7-methyl-1,8-naphthyridin-4(*1H*)-one **15a** and 3-{6-(2-chlorophenyl)-1,2,4-triazolo[3,4-*b*][1,3,4]thiadiazol-3-yl}-1-ethyl-7-methyl-1,8-naphthyridin-4(1*H*)-one **16a**. 

**Figure 9:**
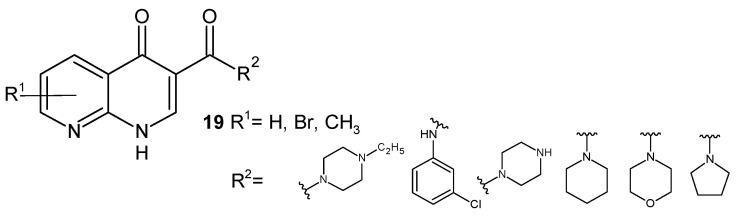
Structures of 1,8-naphthyridine-3-carboxylic acid amides **19**.

V. K. Gurjar et al. [41] synthesized a series of 1,8-naphthyridine-3-carboxylic acid amides, **19** (Figure 9), and assessed their antibacterial potential. These derivatives showed very good bactericidal action toward *E. coli* and a weaker one toward *S. aureus* strains.

**Figure 10:**
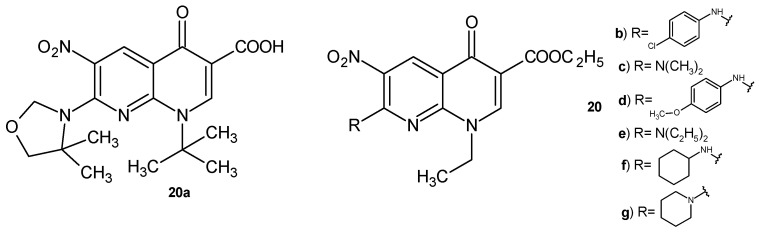
Nalidixic acid derivatives with a 6-nitro group, **20a**–**g**.

Synthesized by Sriram et al. [42], 1-tert-butyl-1,4-dihydro-7-(4,4-dimethyloxazolidin-3-yl)-6-nitro-4-oxo-1,8-naphthyridine-3-carboxylic acid **20a** (Figure 10) showed an antitubercular activity against MDR-TB more potent than isoniazid. Compound **20a** also exhibited high in vivo activity in an animal model, reducing the bacterial load in lung and spleen tissues [42].

N. Suzuki et al. [43] prepared other nalidixic acid derivatives containing a 6-nitro substituent. 7-Amino substituted 1-etyl-1,4-dihydro-6-nitro-4-oxo-1,8-naphthyridine-3-carboxylates were evaluated for their activity against *Trichomonas vaginalis*. Compounds **20b**–**g** (Figure 10) showed the same activity as metronidazole.

**Figure 11:**
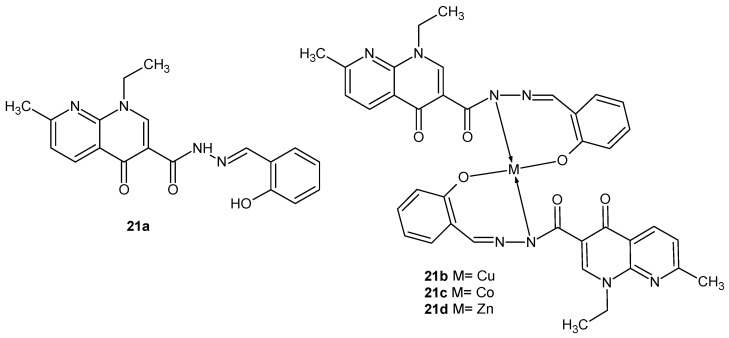
Nalidixic acid hydrazide derivative and its metal complexes, **21a**–**d**.

K.P. Chennam et al. [44] obtained N-(2-hydroxybenzylidene)-1-ethyl-1,4-dihydro-7-methyl-4-oxo-1,8 naphthyridine-3-carbohydrazide **21a** and its complexes, with Cu (II) **21b**, Co (II) **21c**, and Zn (II) **21d** (Figure 11). In vitro antibacterial studies against two Gram-positive (*Bacillus subtilis* and *S. aureus*) and two Gram-negative (*Escherichia coli* and *K. pneumoniae*) strains showed that the activities of metal complexes **21b**–**d** and hydrazide **21a** were comparable to ampicillin, but the most potent was the complex of Cu (II) **21b**. 

**Figure 12:**
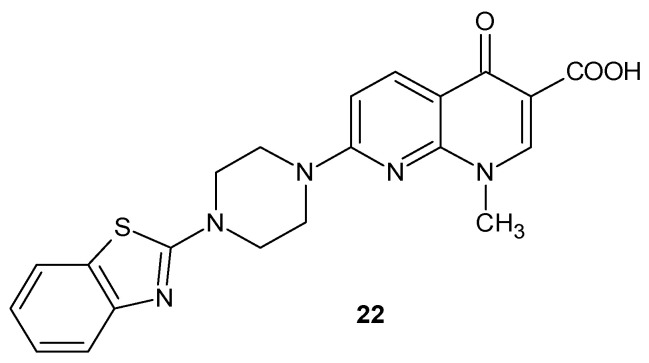
7-[4-(1,3-benzothiazol-2-yl)piperazin-1-yl]-1-methyl-4-oxo-1,4-dihydro-1,8-naphthyridine-3-carboxylic acid **22**.

S. Massari et al. [45] obtained 7-[4-(1,3-benzothiazol-2-yl)piperazin-1-yl]-1-methyl-4-oxo-1,4-dihydro-1,8-naphthyridine-3-carboxylic acid **22** (Figure 12), which showed a very potent and selective anti-HIV activity due to its ability to inhibit Tat-mediated transcription. 

### 2.2. 1,8-Naphthyridine Derivatives with Antimicrobial Activity

**Figure 13:**
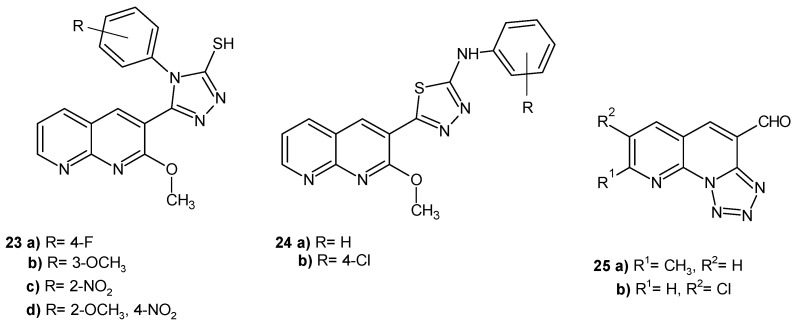
Antibacterial 1,8-naphthyridine derivatives **23**–**25**.

E. Laxminarayana et al. [46] evaluated many 1,8-naphthyridine derivatives for their in vitro antibacterial activity against *S. aureus*, *Bacillus cereus*, *B. subtilis*, *Micrococcus luteus*, *K. pneumoniae*, *Salmonella paratyphi A*, *E. coli*, *Bacillus magaterium*, *Proteus vulgaris*, and *Enterobacter aerogenes*. Some derivatives (Figure 13) exhibited good activity, especially against *P. vulgaris* (**23a**–**d**, **24a**–**b**) and *S. aureus* (**25a**–**b**), but all the compounds were less active than the standard tetracycline.

**Figure 14:**
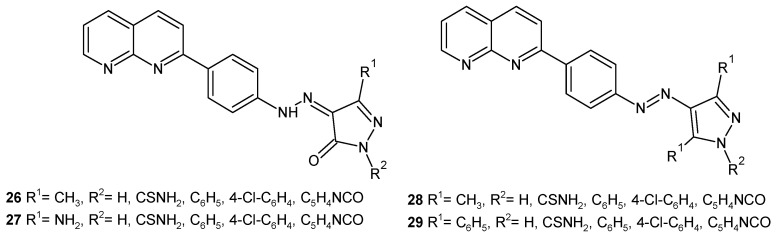
Hydrazono and azo derivatives of 1,8-naphthyridine **26**–**29**.

S. Raja et al. [47] evaluated hydrazono, **26**–**27**, and azo, **28**–**29**, derivatives of 1,8-naphthyridine (Figure 14) for their antibacterial and antifungal activity. The activity was due to the presence of a 4-chloro substituent on the phenyl ring of the pyrazolinone and pyrazole nucleus. Compounds with a 4-chlorophenyl ring, **26**–**29**, were found to be most active against *B. subtilis*, *S. aureus*, *E. coli*, *P. aeruginosa*, *Aspergillus niger*, and *Candida albicans*, comparable to ampicillin and griseofulvin. 

**Figure 15:**
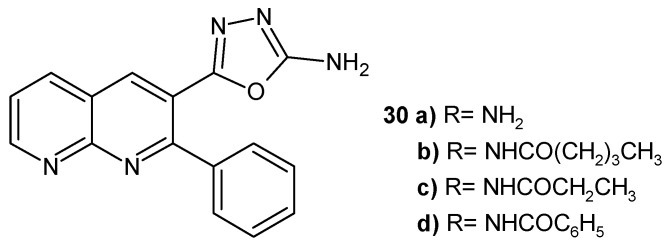
1,8-Naphthyridines with oxadiazole and phenyl ring **30a**–**d**.

Synthesized by K. Md and R. Domala [48], 5-(2-phenyl-1,8-naphthyridine-3-yl)-1,3,4-oxadiazole-2-amine **30a** and its amide derivatives **30b**–**d** (Figure 15) were tested against *S. aureus*, *E. coli*, and *C. albicans*. Propionamide **30c**, pentanamide **30b**, and benzamide **30d** exhibited high antifungal activity. The pentanamide **30b** derivative also showed good antibacterial properties against *Staphylococcus aureus*, comparable to ampicillin.

**Figure 16:**
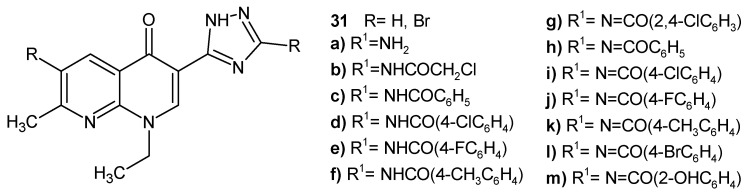
**1**,**8**-Naphthyridinones **31a**–**m**.

N.G. Mohamed et al. [49] obtained 7-methyl-1,8-naphthyridinone derivatives substituted with a 1,2,4-triazole ring, **31a**–**m** (Figure 16), as potential DNA-gyrase inhibitors. Most of the compounds exhibited selective antibacterial activity against *B. subtilis* resistant strains, and some of the obtained compounds were active against *A. actinomycetemcomitans*. The introduction of bromine at C-6 of the naphthyridine scaffold enhanced the antibacterial activity. The tested compounds showed a moderate to high inhibitory effect, having an IC_50_ range 1.7–13.2 µg/mL against DNA gyrase. The most potent were brominated derivatives **31b** and **31f**.

**Figure 17:**
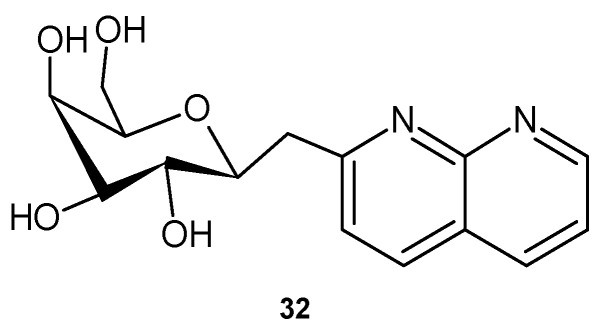
2-(β-D-galactopyranosylmethyl)-1,8-naphthyridine **32**.

2-(β-D-galactopyranosylmethyl)-1,8-naphthyridine **32** (Figure 17), obtained by Nagarajan et al. [50], turned out to be more effective than tetracycline against Gram-negative bacteria such as *K. pneumoniae*, *P. aeruginosa*, and *P. vulgaris*, as well as against *S. aureus.*

**Figure 18:**
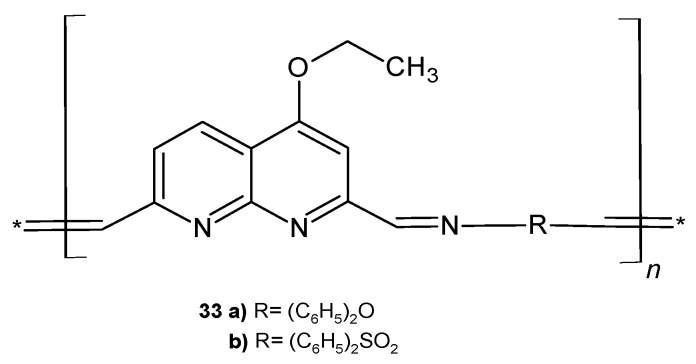
Polymers **33a**–**b**.

M. A. Hussein et al. [51] synthesized a series of heteroaromatic polymers containing 4-ethoxy-2,7-dicarboxyaldehyde-1,8-naphthyridine moieties (Figure 18). Polymers **33a**–**b** showed significant activity against Gram-negative bacteria (*P. aeruginosa*, *Serratia marcescens*, and *E. coli*) compared to ampicillin, and compound **33b** also exhibited a strong antifungal influence against *A. niger* and *Fusarium oxysporum*.

**Figure 19:**
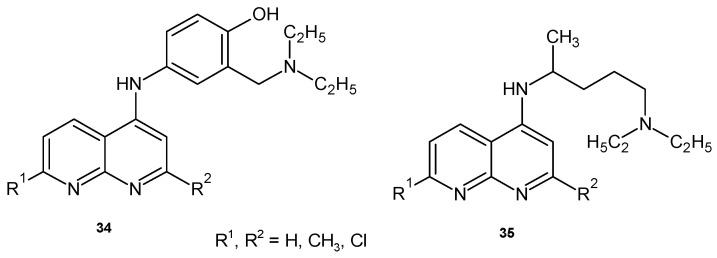
1,8-Naphthyridine derivatives **34**–**35**.

Gordon B. Bavlin and Weng-Lai Tan [52] obtained 5-amino derivatives of 1,8-naphthyridine **34**–**35** (Figure 19), which showed minimal antimalarial activity in vivo against *Plasmodium vinckei vinckei.*

**Figure 20:**
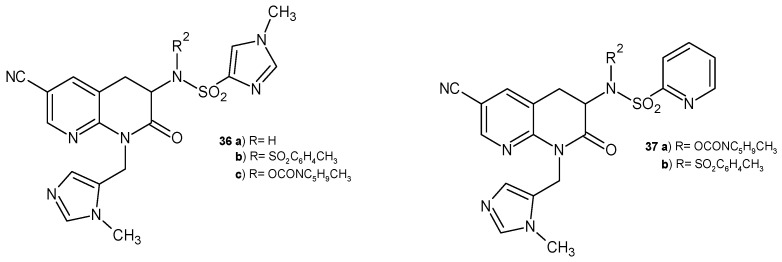
Antimalarial 2-oxo-tetrahydro-1,8-naphthyridine derivatives **36**–**37**.

S. Olepu et al. [53] developed a new class of PFT (protein farnesyltransferase) inhibitors. 6-Cyano-2-oxo-tetrahydro-1,8-naphthyridine derivatives **36**–**37** (Figure 20) selectively inhibited malaria PFT with activities in the low nanomolar range. The resulting compounds were more potent in malaria PFT than in the mammalian enzyme. These derivatives were also more metabolically stable than tetrahydroquinoline.

**Figure 21:**
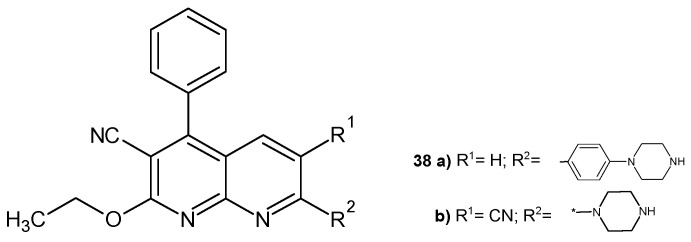
3-Cyano-2-ethoxy-4-phenyl-7-substituted-1,8-naphthyridines **38a**–**b**.

J. M. Quintela et al. [54] obtained 3-cyano-2-ethoxy-4-phenyl-7-substituted-1,8-naphthyridines and submitted them to in vitro tests for antiparasitic activity against the ciliates *Philasterides dicentrarchi.* The presence of the piperazine ring was found to be essential for strong antiprotozoal activity. The most active compounds were **38a**–**b** (Figure 21), whose activity was comparable to that of norfloxacin. 

**Figure 22:**
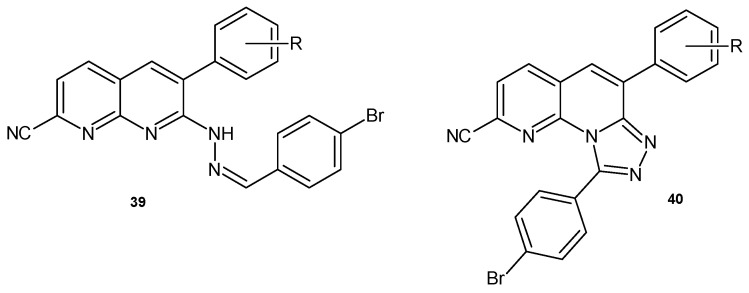
6-Aryl-1,8-naphthyridine-2-carbonitrile derivatives **39**–**40**.

K.K. Priya et al. [55] prepared 7-(2-(4-bromobenzylidene)hydrazinyl)-6-aryl-1,8-naphthyridine-2-carbonitrile **39** and 6-aryl-[1,2,4]triazolo [4,3-*a*][1,8]naphthyridine **40** derivatives (Figure 22), which were evaluated for their antimicrobial activity against bacterial strains (*B. megaterium*, *M. luteus*, *S. typhi*, and *E. coli*) and fungal strains (*A. niger*, *Aspergillus flavus*). All compounds showed good levels of activity, comparable to reference drugs (streptomycin and nystatin).

**Figure 23:**
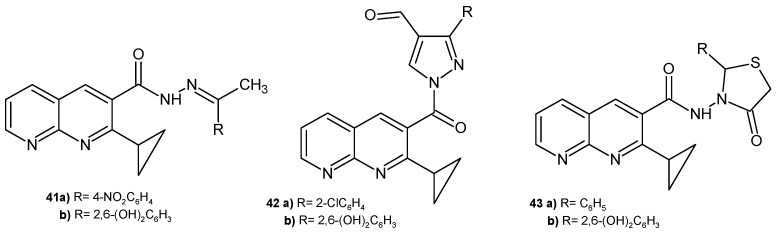
2-Cyclopropyl-1,8-naphthyridine derivatives with antibacterial activity.

A. Narender et al. [56] prepared 2-cyclopeopyl-1,8-naphthyridine derivatives, which were tested in vitro against *S. aureus*, *E. coli*, *K. pneumoniae*, *S. paratyphi A*, *S. paratyphi B*, and *M. luteus.* The most active derivatives (growth inhibition zones were 6–16 mm at a concentration of 1 mg/mL) were compounds **41a**–**b**, **42a**–**b**, and **43a**–**b** (Figure 23).

**Figure 24:**
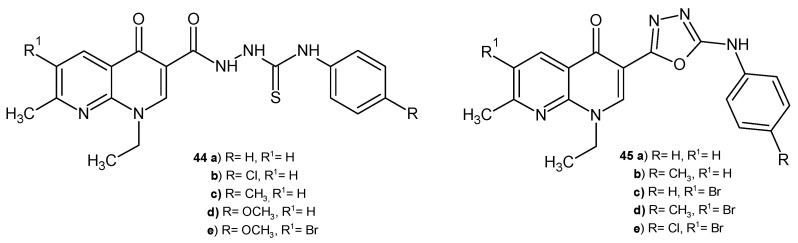
Thiosemicarbazides and 1,3,4-oxadiazoles of 1,8-naphthyridine **44**–**45**.

F.A. Omar et al. [57] synthesized a series of 1,8-naphthyridine-3-thiosemicarbazides, **44**, and 1,8-naphthyridine-3-(1,3,4-oxadiazoles), **45** (Figure 24), as potential DNA gyrase inhibitors. These derivatives **44**–**45** were screened for antibacterial activity against *S. aureus*, *B. cereus*, *E. coli*, *K. pneumoniae*, *P. aeruginosa*, and *Mycobacterium smegmatis*. The tested compounds were not active against *P. aeruginosa.* The highest activity against *S. aureus* was shown by compounds **44a**–**b**, **45a**–**b** (MIC values in the range of 6–7 mM), while the most active against *M. smegmatis* were derivatives **44b**–**d**, **45c**–**e** (MIC values in the range of 5.4–7.1 mM). The bromination of the naphthyridine skeleton resulted in better antibacterial activity against *B. cereus*. Molecular docking and a DNA gyrase inhibition assay showed that derivatives **44b**, **44e**, **45a**, and **45d** were the most active, comparable to nalidixic acid. 

**Figure 25:**
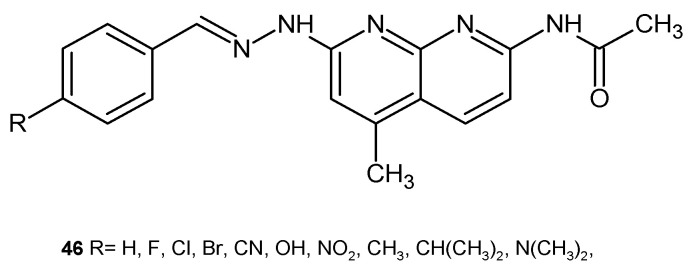
*N*-benzylidene-*N*′-(5-methyl-2-acetamido[1,8]naphthyridin-7-yl)hydrazine derivatives **46**.

G. Turan-Zitouni et al. [58] evaluated the obtained *N*-benzylidene-*N*′-(5-methyl-2-acetamido [1,8]naphthyridin-7-yl)hydrazine derivatives **46** (Figure 25) for antimicrobial activity against *M. luteus*, *B. subtilis*, *Salmonella typhimirium*, *S. aureus*, *E. coli*, *Listeria monocytogenes*, and *C. albicans*. Tested compounds **46** showed an antibacterial activity comparable to streptomycin only against *S. typhimirium*. All of these derivatives were also effective against *C. albicans* compared to ketoconazole.

**Figure 26:**
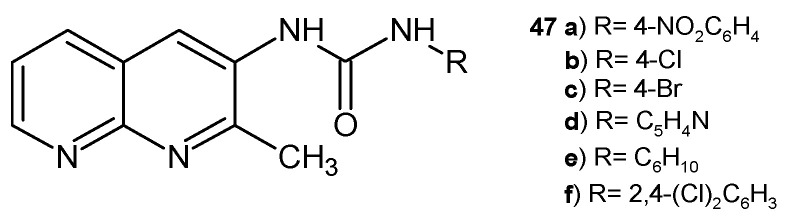
3-(2-Methyl-1,8-naphthyridin-3-yl)ureas **47**.

D. Ramesh et al. [59] synthesized a series of 3-(2-methyl-1,8-naphthyridin-3-yl) ureas, **47** (Figure 26), as potential antifungal agents. The obtained compounds were found to be active against the following fungal strains: *Alternaria alternata* **47a**–**d**, *F. oxysporum* **47a**,**b**,**e**, and *Curvularia lunata* **47a**,**b**,**f**.

**Figure 27:**
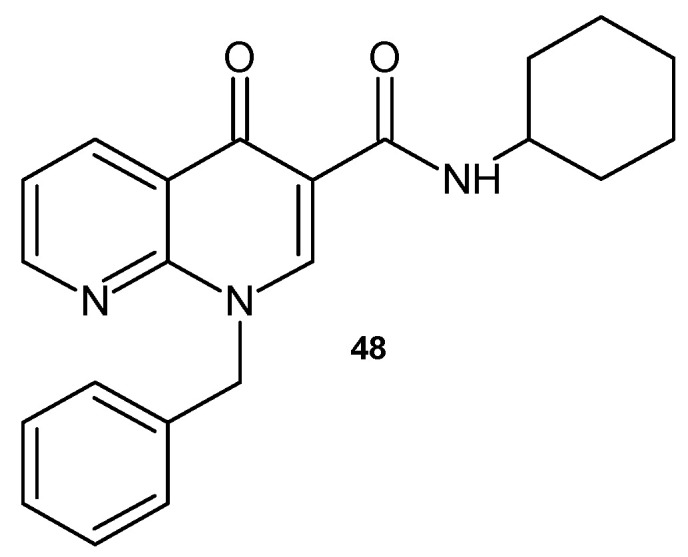
1-Benzyl-*N*-cyclohexyl-4-oxo-1,4-dihydro-1,8-naphthyridine-3-carboxamide **48**.

V.K. Gurjar et al. [60] obtained 1-benzyl-*N*-cyclohexyl-4-oxo-1,4-dihydro-1,8-naphthyridine-3-carboxamide **48** (Figure 27) as an effective antimicrobial agent similar to ciprofloxacine.

**Figure 28:**
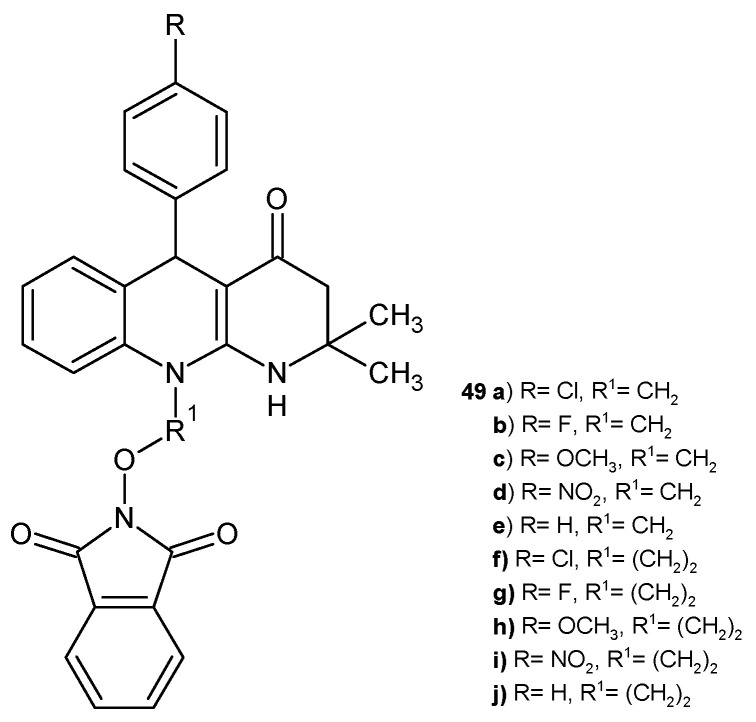
Benzo[*b*][1,8]naphthyridine derivatives **49a**–**j**.

D. Bhambi et al. [61] obtained tetrahydrobenzo[*b*][1,8]naphthyridine derivatives substituted with a phthalimidoxy group, **49a**–**j** (Figure 28). All compounds were evaluated for antimicrobial activity against *Proteus mirabilis*, *B. subtilis*, *K. pneumonia*, *E. coli*, *S. albicans*, and *Aspergillus fumigatus* and showed moderate antibacterial activity but strong antifungal efficacy. 

**Figure 29:**
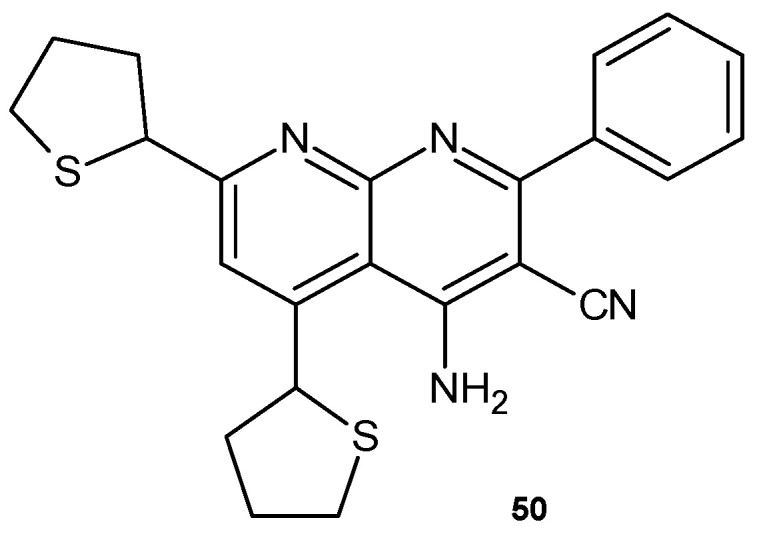
4-Amino-2-phenyl-5,7-di(thien-2-yl)-1,8-naphthyridine-3-carbonitrile **50**.

4-Amino-2-phenyl-5,7-di(thien-2-yl)-1,8-naphthyridine-3-carbonitrile **50** (Figure 29), obtained by E. Mohamed et al. [62], was evaluated for its antimicrobial activity against *E. coli*, *B. subtilis*, *A. flavus*, and *C. albicans* and showed no high antibacterial activity but better antifungal activity against *A. flavus*.

**Figure 30:**
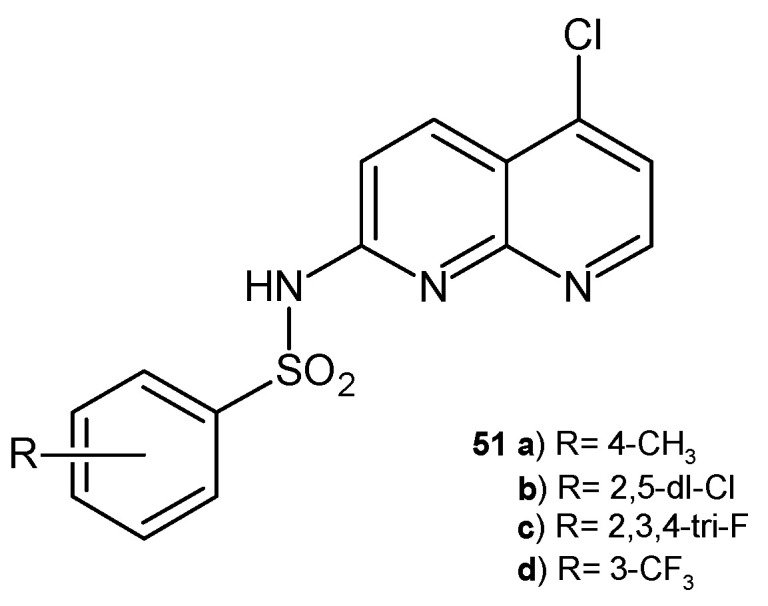
1,8-Naphthyridine sulphonamides **51a**–**d**.

C. Oliveira-Tintino et al. [63] investigated the antibacterial activity of 1,8-naphthyridinesulphonamides. The tested compounds did not show direct antibacterial activity but effectively reduced the MIC of multidrug-resistant bacteria by associating with ethidium bromide and norfloxacin. Additionally, molecular docking studies showed that 1,8-naphthyridinesulfonamides **51a**–**d** (Figure 30) can attenuate the resistance of *S. aureus* via molecular mechanisms related to the inhibition of the NorA efflux pump [63].

**Figure 31:**
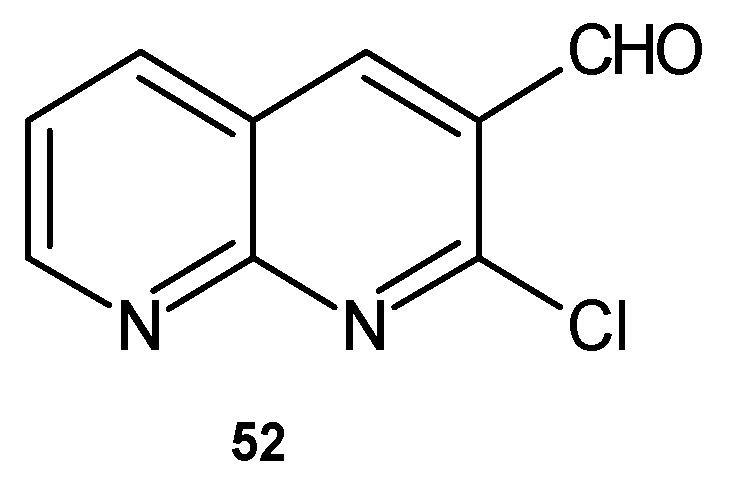
2-Chloro-1,8-naphthyridine-3-carbaldyhyde **52**.

K. M. Fayyadh et al. [64] evaluated 2-chloro-1,8-naphthyridine derivatives for their antibacterial activity. Among the tested compounds, 2-chloro-1,8-naphthyridine-3-carbaldyhyde **52** (Figure 31) showed moderate activity against *E. coli* and high activity against *S. pyogenes*.

**Figure 32:**
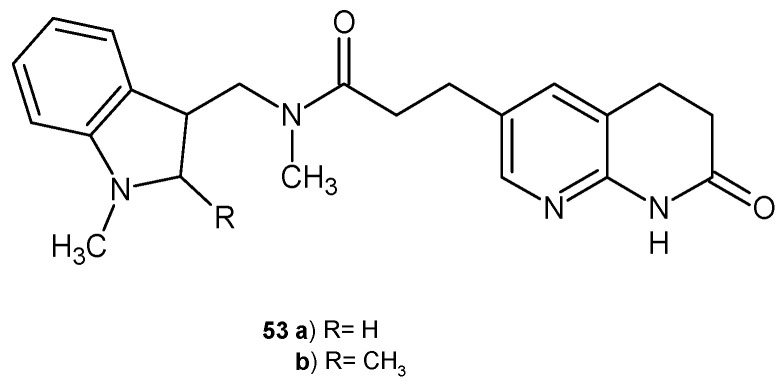
Naphthyridin-2-one derivatives **53** as FabI and FabK inhibitors.

M. A. Seefeld et al. [65] obtained a series of naphthyridin-2-one derivatives with an indole moiety as inhibitors of bacterial enoyl-ACP reductases FabI and FabK. The most active derivatives, **53a**–**b** (Figure 32), were found to be potent FabI and FabK inhibitors. Additionally, compound **53b** showed good in vivo efficacy after oral administration in a rat model of infection with a multidrug-resistant strain of *S. aureus*.

**Figure 33:**
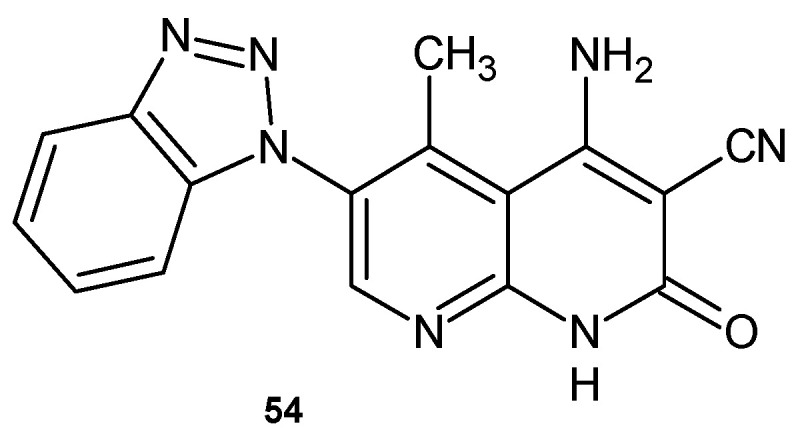
4-Amino-6-benzotriazol-1-yl-1,2-dihydro-5-methyl-2-oxo-1,8-naphthyridine-3-carbonitrile **54**.

4-Amino-6-benzotriazol-1-yl-1,2-dihydro-5-methyl-2-oxo-1,8-naphthyridine-3-carbonitrile **54** (Figure 33), obtained by F. Al-Omran et al. [66], showed strong bactericidal and fungicidal activity against *S. aureus* and *A. niger* but was inactive against *E. coli*, *B. subtilis*, and *F*. *oxysporium*.

**Figure 34:**
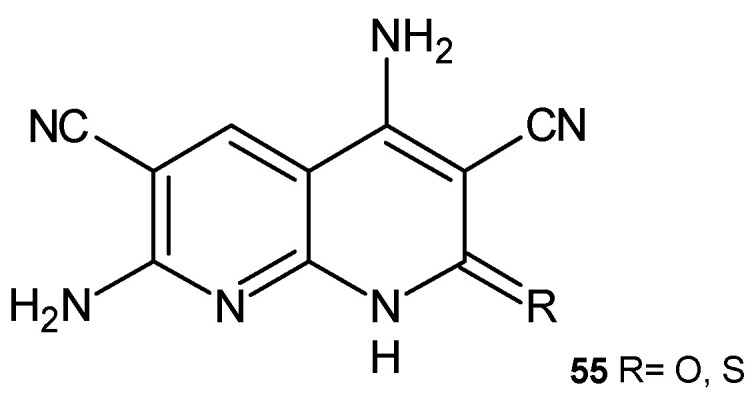
4,7-Diamino-2-oxo/thioxo-1,2-dihydro[1,8]naphthyridine-3,6-dicarbonitrile, **55**.

4,7-Diamino-2-oxo-1,2-dihydro-1,8-naphthyridine-3,6-dicarbonitrile and 4,7-diamino-2-thioxo-1,2-dihydro-1,8-naphthyridine-3,6-dicarbonitrile, **55** (Figure 34), synthesized by El-Remaily et al. [67], showed moderate activity against *Staphylococcus albus*. Compounds **55** were found to be highly toxic to *Artemia salina* larvae.

**Figure 35:**
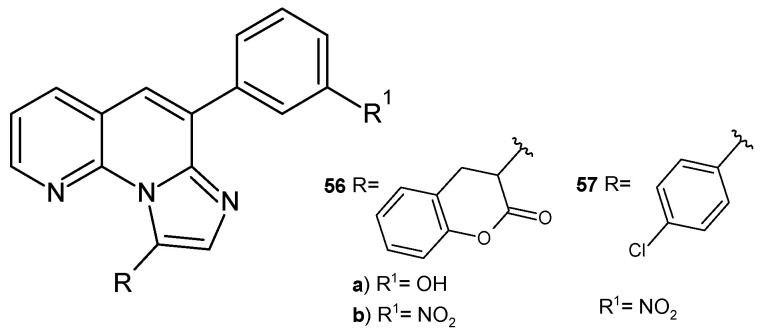
Imidazo [1,2-*a*][1,8]naphthyridine derivatives **56**–**57**.

S. Banoth et al. [68] prepared imidazo[1,2-*a*][1,8]naphthyridine derivatives **56**–**57** (Figure 35). The obtained compounds were evaluated for their antibacterial and antifungal activity. Compounds **56a**–**b** and **57** demonstrated high activity against *S. aureus*, *E. coli*, *Candida metapsilosis*, and *A. niger*, comparable to that of penicillin and griseofulvin. 

**Figure 36:**
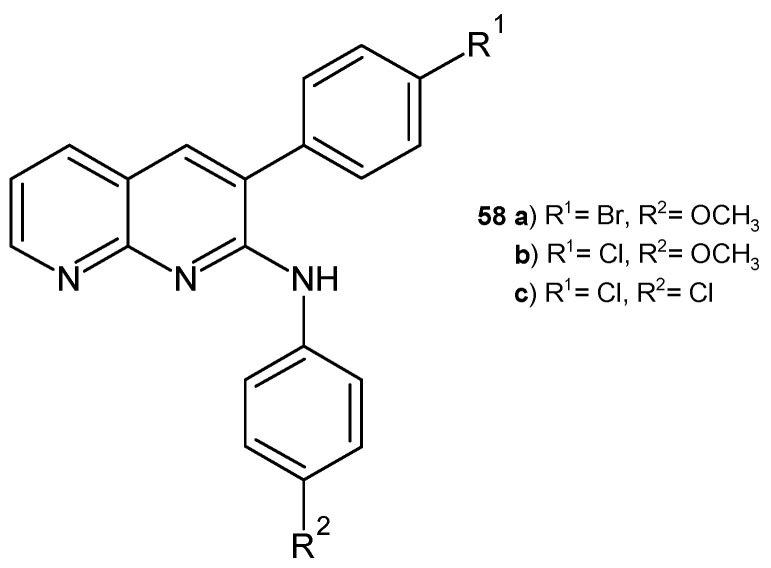
*N*-3-diaryl-1,8-naphthyridin-2-amine derivatives **58a**–**c**.

A series of *N*-3-diaryl-1,8-naphthyridin-2-amines were synthesized by D. Ravi et al. [69] and evaluated for their antibacterial activity against *B. subtilis* and *E. coli* using the filter paper disc technique. The most active, similar to streptomycin, were derivatives **58a**–**c** (Figure 36). 

**Figure 37:**
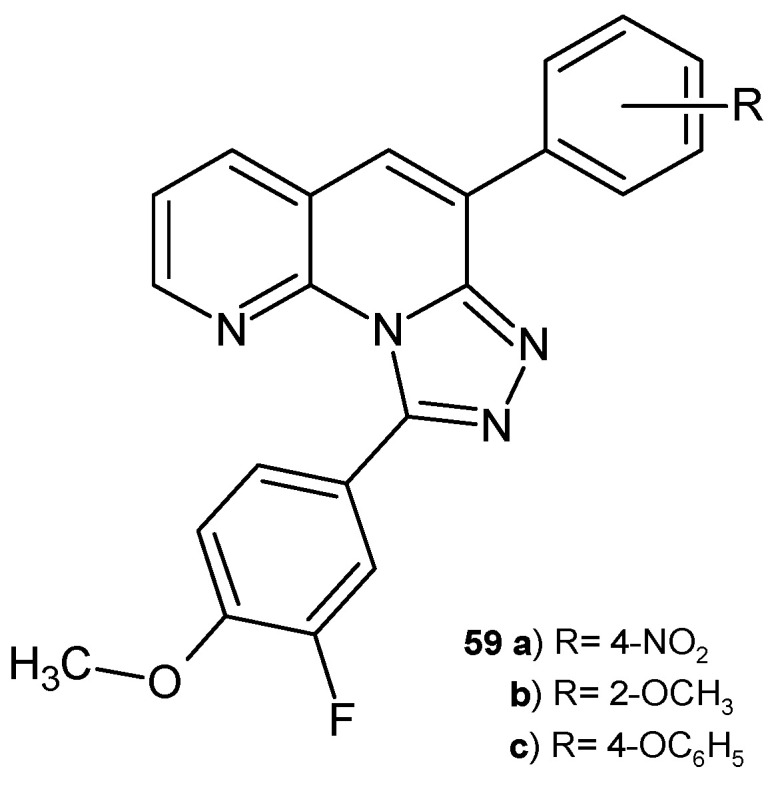
9-(3-Fluoro-4-methoxyphenyl)-6-aryl-[1,2,4]triazolo [4,3-a][1,8]naphthyridine derivatives **59a**–**c**.

B. Sakram et al. [70] synthesized 9-(3-fluoro-4-methoxyphenyl)-6-aryl-[1,2,4]triazolo [4,3-*a*][1,8]naphthyridine derivatives. These compounds were screened in vitro for their antibacterial activity against *S. aureus B. subtilis*, *E. coli*, and *K. pneumoniae* and for antifungal activity against *A. flavus* and *F. oxysporum*. Derivatives **59a**–**c** (Figure 37) showed maximal inhibition zones against all tested microorganisms compared to ciprofloxacin and amphotericin B. Molecular docking studies demonstrated the maximum interaction of the tested compounds **59a**–**c** with His228.

**Figure 38:**
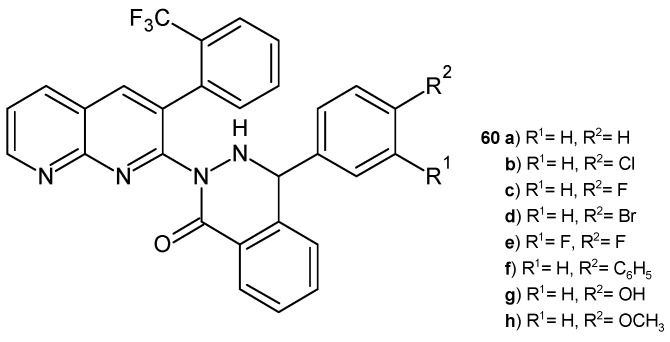
4-Aryl-2-(3-(2-(trifuoromethyl)phenyl)-1,8-naphthyridin-2-yl)phthalazin-1(2*H*)-ones **60a**–**h**.

B. Sakram et al. [71] obtained 4-aryl-2-(3-(2-(trifluoromethyl)phenyl)-1,8-naphthyridin-2-yl)phthalazin-1(2*H*)-ones **60a**–**h** (Figure 38). Some of the compounds **60a**–**d** showed good antibacterial and antifungal activity. Derivatives containing 4-OCH_3_ and 4-OH substitution in the benzene ring exhibited better antimicrobial activity (the growth inhibition zones were 15–19 mm at a concentration of 250 ppm).

**Figure 39:**
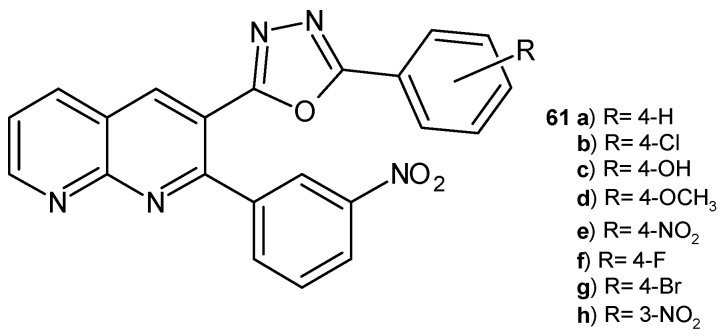
2-(2-(3-Nitrophenyl)-1,8-naphthyridin-3-yl)-5-phenyl-1,3,4-oxadiazoles derivatives **61a**–**h**.

2-(2-(3-Nitrophenyl)-1,8-naphthyridin-3-yl)-5-phenyl-1,3,4-oxadiazoles derivatives **61a**–**h** (Figure 39) were synthesized by B. Sakram et al. [72]. These compounds were evaluated for their antibacterial and antifungal activity in vitro. The most active against *S. aureus*, *E. coli*, *A. niger*, and *C. metapsilosis* were the 4-hydroxy **61c** and 4-fluoro **61f** derivatives. 

**Figure 40:**
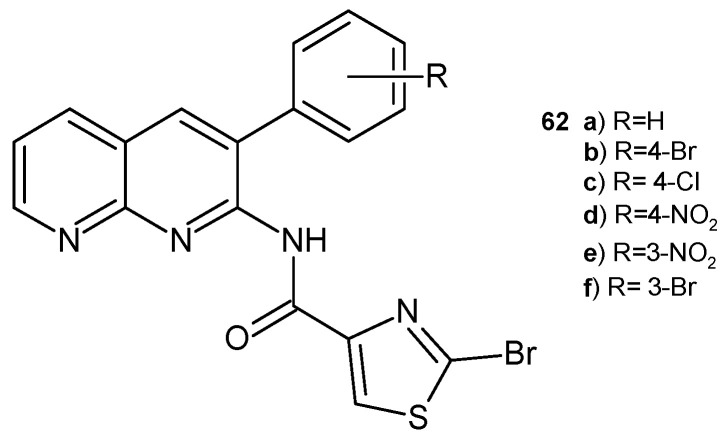
2-Bromo-*N*-(3-aryl-1,8-naphthyridin-2-yl)thiazole-4-carboxamide derivatives **62a**–**f**.

2-Bromo-*N*-(3-aryl-1,8-naphthyridin-2-yl)thiazole-4-carboxamide derivatives **62a**–**f** (Figure 40), synthesized by B. Sonyanaik et al. [73], were screened for their in vitro antibacterial activity against *S. aureus* and *E. coli*, as well as for their antifungal activity against pathogenic fungal strains *A. niger* and *A. flavus.* Compounds **62a**–**b** showed high antimicrobial efficacy. Zones of inhibition (in mm) were comparable to those of griseofulvin and ampicillin. 

**Figure 41:**
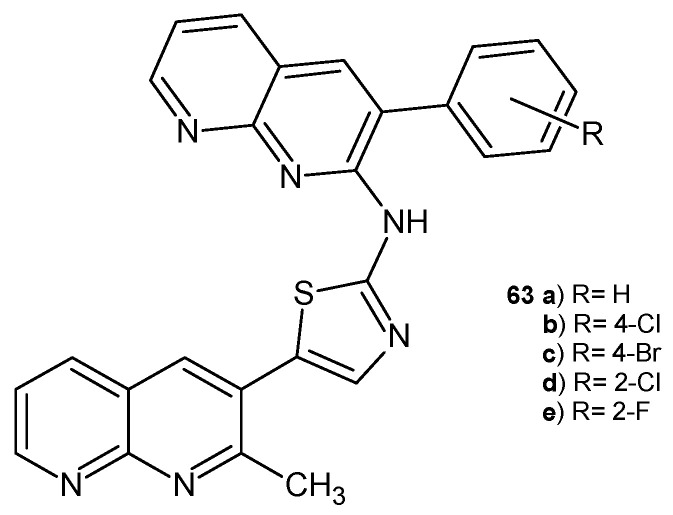
*N*-(3-aryl-1,8-naphthyridin-2-yl)-5-(2-methyl-1,8-naphthyridin-3-yl)thiazol-2-amine derivatives **63a**–**e**.

N-(3-aryl-1,8-naphthyridin-2-yl)-5-(2-methyl-1,8-naphthyridin-3-yl)thiazol-2-amine derivatives **63a**–**e** (Figure 41), synthesized by A. Ashok et al. [74], were evaluated for their in vitro antibacterial and antifungal activity against *S. aureus*, *E. coli*, *A. niger*, and *C. albicans*. Compounds containing a chloro substituent, **63b** and **63d**, displayed the highest activity when compared with penicillin or griseofulvin (MIC values in the range of 35.5–75.5 μg/mL). 

**Figure 42:**
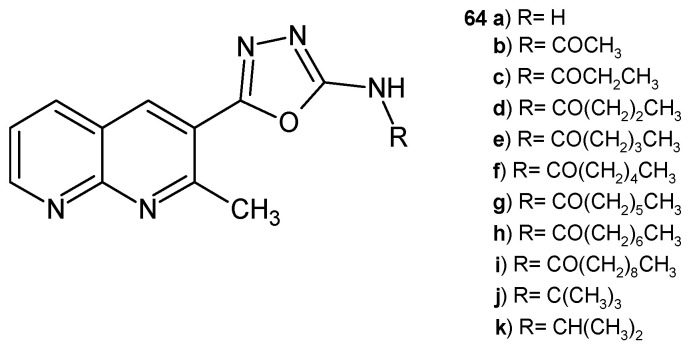
5-(2-methyl-1,8-naphthyridin-3-yl)-1,3,4-oxadiazol-2-amines **64a**–**k**.

Kumar Parangi et al. [75] synthesized 5-(2-methyl-1,8-naphthyridin-3-yl)-1,3,4-oxadiazol-2-amine derivatives **64a**–**k** (Figure 42), which were screened for their in vitro antibacterial and antifungal activity against strains of *S. aureus*, *E. coli*, and *C. albicans*. Compound **64b** showed high activity against *S. aureus* (the 1 mm zone at the concentration of 100 µg/mL), similar to ampicillin. Derivatives **64b** and **64i** demonstrated moderate properties against *E. coli*. Compounds **64a**, **64f**, and **64i** showed significant antifungal activity compared to ketoconazole. Molecular docking studies showed that compounds **64b**, **64f**, **64g**, **64i**, **64j**, and **64k** interacted with the target protein more efficiently [75]. 

**Figure 43:**
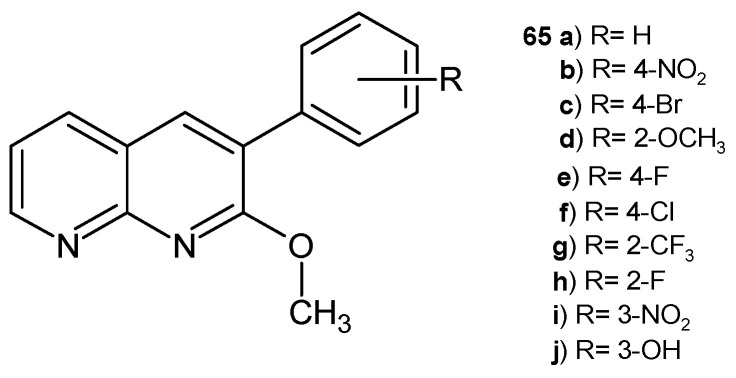
2-Methoxy-3-aryl-1,8-naphthyridine derivatives **65a**–**j**.

D. Ravi et al. [76] obtained 2-methoxy-3-aryl-1,8-naphthyridines **65a**–**j** (Figure 43), which were evaluated for their antimicrobial activity against bacterial strains *P. vulgaris*, *S. typhimurium*, *E. aerogenes*, and *S. aureus* and fungi strains *Malassezia pachydermatis* and *C. albicans*. The compound with fluorine substitution at the para position of the phenyl ring, **65e**, and the derivative with a trifluoromethyl group, **65g**, exhibited very good antimicrobial activity against the tested strains (MIC = 35–125 µg/mL). Based on the molecular docking results, compounds **65e** and **65g** showed the highest docking and hydrogen-bonding score and a good affinity towards the DNA gyrase receptor.

**Figure 44:**
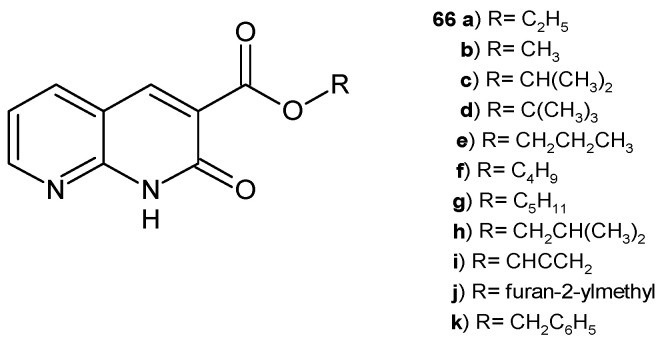
2-Oxo-1,2-dihydro-1,8-naphthyridine-3-carboxylates **66a**–**k**.

B. Sakram et al. [77] obtained 2-oxo-1,2-dihydro-1,8-naphthyridine-3-carboxylates **66a**–**k** (Figure 44), which were screened for their antimicrobial activity against *S. pyogenes*, *E. coli*, *Saccharomyces cerevisiae*, and *Aspergillus terreus* by the agar well diffusion method, using ciprofloxacin and nystatin as standards. Compounds **66b**, **66i**, and **66j** showed the highest antibacterial and antifungal activity, similar to the reference drugs.

**Figure 45:**
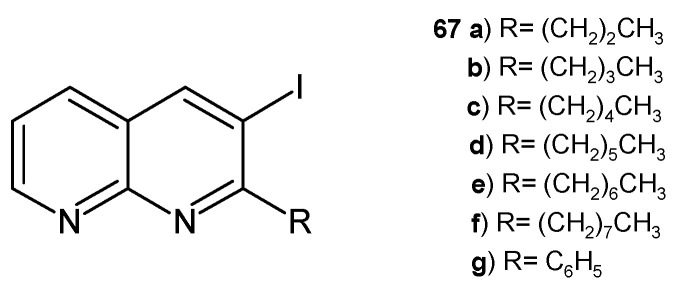
3-Iodo-1,8-naphthyridine derivatives **67a**–**g**.

The same researchers [78] synthesized 3-iodo-1,8-naphthyridines **67a**–**g** (Figure 45), which were evaluated for their antimicrobial activity against *S. aureus*, *B. subtilis*, *E. coli*, *K. pneumoniae*, *A. flavus*, and *F. oxysporum*. All the tested compounds showed good antibacterial and antifungal properties, but the most potent derivatives, **67d** and **67g**, exhibited maximum zones of inhibition against the tested strains as compared to ciprofloxacin and amphotericin B.

**Figure 46:**
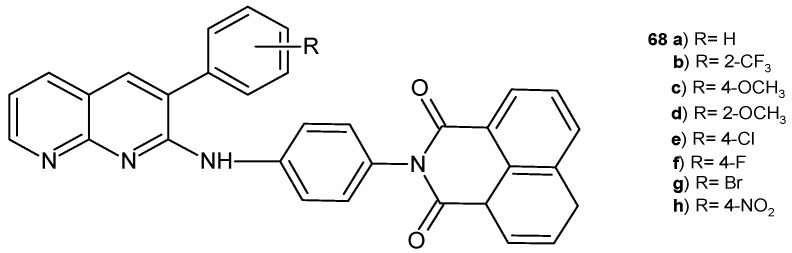
2-{4-[(3-Aryl-1,8-naphthyridin-2-yl)amino]-phenyl}-1*H*-benzo[*de*]isoquinoline-1,3(2*H*)-diones **68a**–**h**.

A series of 2-{4-[(3-aryl-1,8-naphthyridin-2-yl)amino]-phenyl}-1*H*-benzo[*de*]isoquinoline-1,3(2*H*)-dione derivatives, **68a**–**h** (Figure 46), was obtained by B. Sakram et al. [79] and screened for their antimicrobial activity against *E. coli*, *B. subtilis*, *K. pneumoniae*, and *S. aureus*. The tested compounds showed moderate activity, and only derivatives with the trifluoro group, **68b**, exhibited high activity against *B. subtilis*, exhibiting a maximum zone of inhibition close to that of chloramphenicol.

**Figure 47:**
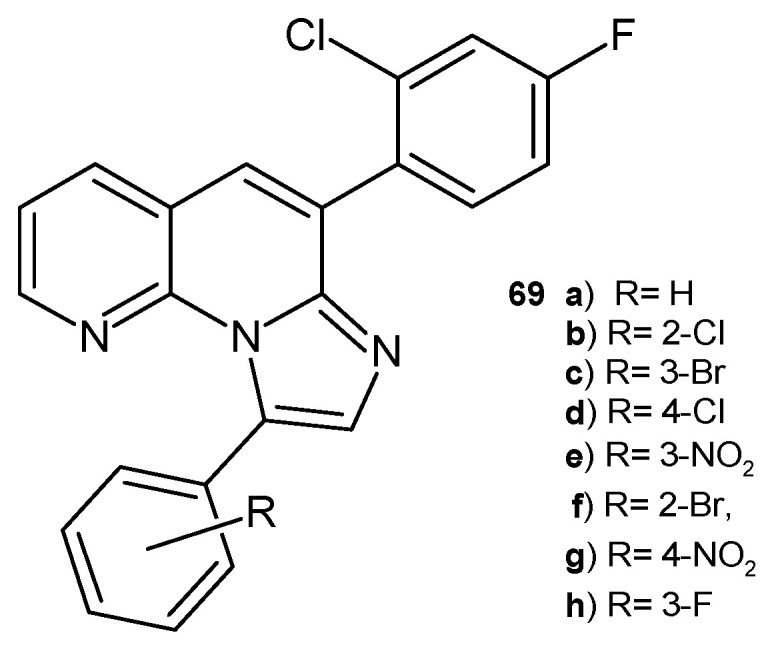
6-(2-Chloro-4-fluorophenyl)-9-phenylimidazo[1,2-*a*][1,8]naphthyridines **69a**–**h**.

B. Sonyanaik et al. [80] synthesized 6-(2-chloro-4-fluorophenyl)-9-phenylimidazo [1,2-*a*][1,8]naphthyridine derivatives **69a**–**h** (Figure 47), which were evaluated for their in vitro antibacterial activity against *S. aureus* and *E. coli* and antifungal activity against *A. niger* and *C. metapsilosis* using the agar diffusion method. All the tested compounds showed good antimicrobial activity, but the most potent was compound **69d**, with properties similar to that of the reference drugs (penicillin and clotrimazole). Molecular docking studies confirmed the data on antimicrobial activity.

**Figure 48:**
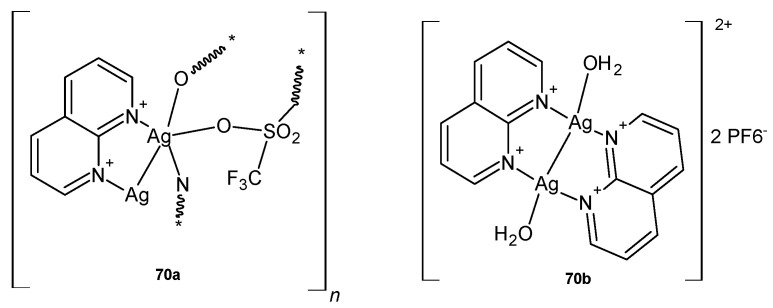
Silver(I) complexes with 1,8 naphthyridine **70a**–**b**.

Silver(I) complexes with 1,8-naphthyridine, **70a**–**b** (Figure 48), were prepared by D. P. Aśanin et al. [81] and biologically evaluated as potential antimicrobial agents. The tested complexes showed significant in vitro activity against *S. aureus*, *P. aeruginosa*, and *Candida* species (with MIC values in the range of 1.56–7.81 µg/mL) and low in vivo toxicity in the *C. elegans* nematode model.

**Figure 49:**
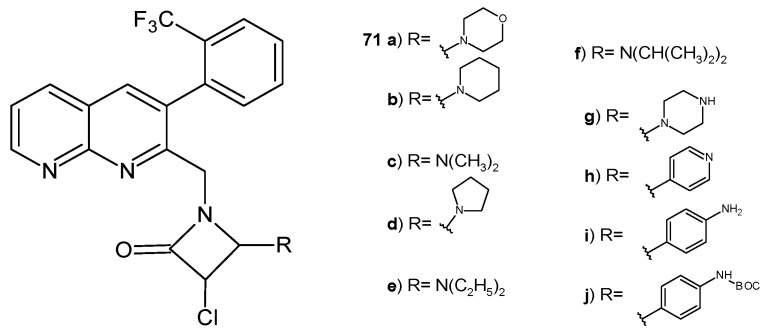
3-Chloro-1-((3-(2-(trifluoromethyl)phenyl)-1,8-naphthyridin-2-yl)amino)azetidin-2-ones **71a**–**j**.

K. M. Pandaya et al. [82] obtained β-lactam derivatives of 1,8-naphthyridines, **71a**–**j** (Figure 49), and assessed their antimicrobial activity. 3-Chloro-1-((3-(2-(trifluoromethyl)phenyl)-1,8-naphthyridin-2-yl)amino)azetidin-2-one derivatives **71a**, **71d**, **71g**, **71i**, and **71j** were found to have a good efficacy against *S. pneumoniae*, *Clostridium tetani*, *S. typhi*, and *Vibrio cholera* comparable to ampicillin and ciprofloxacin. Derivatives **71a**, **71b**, **71d**, and **71g** showed promising antitubercular activity, with MIC values of 6.45 µg/mL, 6.14 µg/mL, 4.19 µg/mL, and 3.11 µg/mL, respectively [82]. 

**Figure 50:**
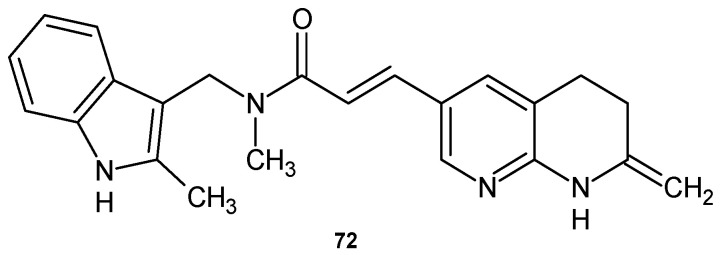
N-methyl-N-(2-methyl-1*H*-indol-3-ylmethyl)-3-(7-oxo-5,6,7,8-tetrahydro-1,8-naphthyridin-3-yl)acrylamide **72**.

N-methyl-N-(2-methyl-1*H*-indol-3-ylmethyl)-3-(7-oxo-5,6,7,8-tetrahydro-1,8-naphthyridin-3-yl)acrylamide **72** (Figure 50) showed excellent activity against multidrug-resistant strains of *S. aureus* and *S. epidermidis* and excellent in vivo efficacy in a rat model of *S. aureus* infection [83]. Compound **72** exhibited good FabI inhibitory activity and weak FabK inhibitory activity. Derivative **72** also showed activity against a broader range of Gram-negative bacteria. Studies indicate that this compound is a substrate for the *H. influenzae* efflux pump [83].

**Figure 51:**
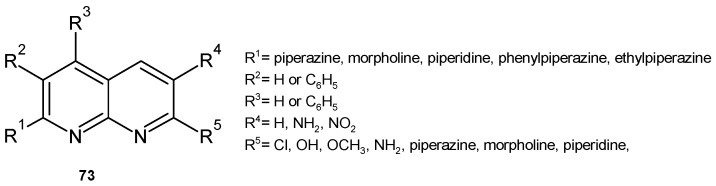
1,8-Naphthyridine derivatives **73**.

P. M. Sivakumar et al. [84] investigated the influence of substituents in the 3- or 4-phenyl-1,8-naphthyridine scaffold, **73** (Figure 51), on the antitubercular and antibacterial activity of these derivatives. The piperidinyl moiety at position 2 or 7 enhanced the antitubercular activity. Morpholinyl substitution at these positions resulted in weaker activity. 7-Amino, 7-chloro, or 7-methoxy derivatives showed moderate to good antimycobacterial activity, but the 6-amino or 6-nitro derivatives were found to be inactive. However, the 6-nitro substituent resulted in the highest activity against *S. aureus*, and 7-amino derivatives exhibited very good activity against *E. coli*. QSAR results showed that compounds with a large number of hydrogen bond donors and a low heat of formation and solvent-accessible surface area are effective antibacterial agents. Most of the active compounds showed a positive correlation, while the least active compounds showed a negative correlation between antitubercular activity and logP [84].

**Figure 52:**
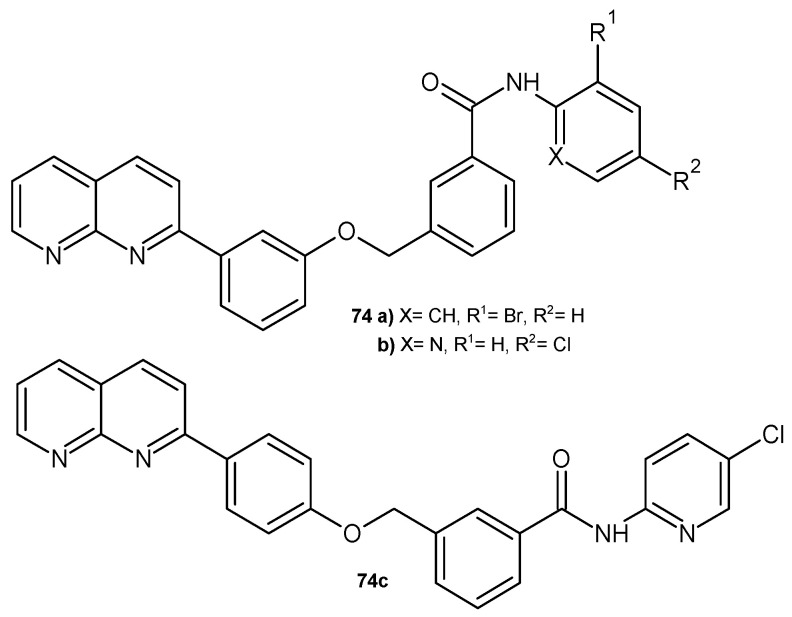
2-Aryl-1,8-naphthyridine derivatives **74a**–**c**.

2-Aryl-1,8-naphthyridine derivatives **74** (Figure 52), synthesized by G. V. Bhasker et al. [85], were evaluated for their antibacterial activity against *E. coli*, *S. aureus*, *K. pneumonia*, and *B. subtilis*. All the tested compounds were inactive against *B. subtilis*. In vitro screening results showed that some compounds, **74a**–**c**, exhibited antibacterial activity against *E. coli* and *S. aureus* with zones of inhibition of 4–6 mm.

### 2.3. Polycyclic Derivatives of 1,8-Naphthyridine

**Figure 53:**
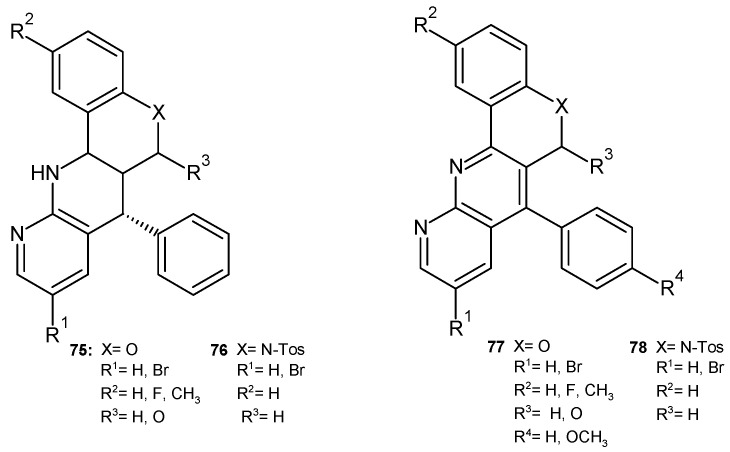
Fused naphthyridines **75**–**78**.

E. Melcón-Fernandez et al. [86] synthesized and evaluated the antileishmanial activity of 1,8-substituted fused naphthyridines **75**–**78** (Figure 53) in in vitro and ex vivo tests against *Leishmania infantum*. The presence of a nitrogen atom in the naphthyridine-fused ring (compounds **76** and **78**) is important for the increased biological activity. Compounds **75** and **77** showed significant activity against leishmaniasis but also high toxicity.

**Figure 54:**
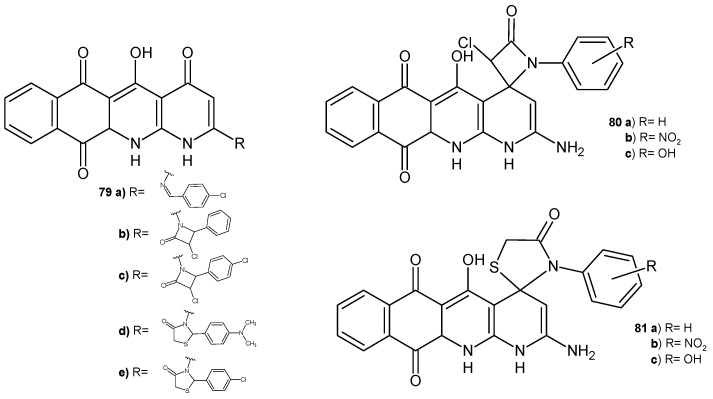
Naphtho[2,3-*b*][1,8]naphthyridine derivatives **79**–**81**.

A series of naphtho[2,3-*b*][1,8]naphthyridine derivatives **79**–**81** (Figure 54) synthesized by Elkanzi [87] were tested in vitro for their antibacterial and antifungal activity. Compounds **79a**, **79e**, **80a**, **82b** displayed the highest activity against *S. aureus*, and compounds **79c**, **80b**, **81b** exhibited very good activity against *B. subtilis* and *E. coli* compared to ampicillin. Most of these compounds displayed good activity, but the presence of a chloro or nitro group at the para position of the phenyl ring improved their efficiency. Compounds **79a**, **79b**–**c**, **79d**–**e**, **80b**–**c**, and **81a**–**c** displayed potent antifungal activity against *A. flavus* and *C. albicans* similar to that of amphotericin B. Molecular docking studies showed that the most active antimicrobial compounds have good energy-binding properties in the active site of topoisomerase II [87].

**Figure 55:**
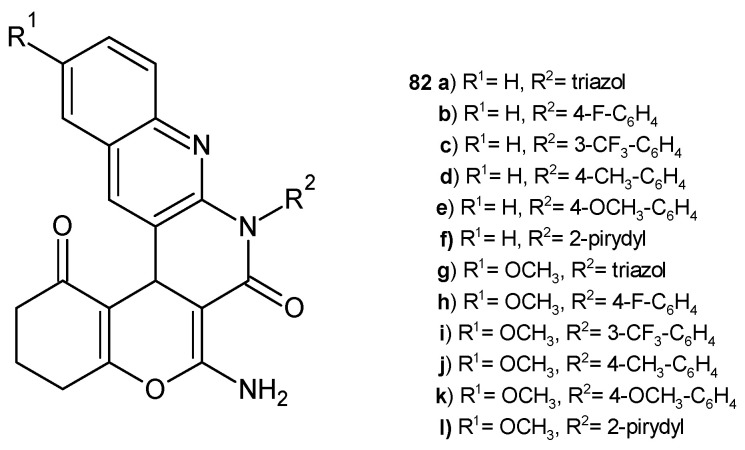
Chromeno-1,8-naphthyridine derivatives **82a**–**l**.

A series of chromeno-1,8-naphthyridine derivatives, **82** (Figure 55), prepared by J. D. Gohil et al. [88], was evaluated for their antimicrobial activity against bacterial strains *S. pneumonia*, *B. subtilis*, *C. tetani*, *E. coli*, *S. typhi*, and *V. cholera* and fungal strains *C. albicans* and *A. fumigatus* using the broth microdilution method. The obtained compounds showed moderate antifungal properties. The presence of fluorine increased the antibacterial activity of the tested compounds. The most potent were derivatives **82b**–**c** and **82h**–**i** (MIC = 62.5–200 µg/mL). Compounds with a pyridine moiety also showed better antibacterial activity. Compound **82l** exhibited excellent activity against all Gram-positive bacteria strains tested (MIC = 62.5–100 µg/mL), and compound **82f** was strongly active against *C. tetani* (MIC = 62.5 µg/mL).

### 2.4. 1,8-Naphthyridine Conjugated with Ketolide

**Figure 56:**
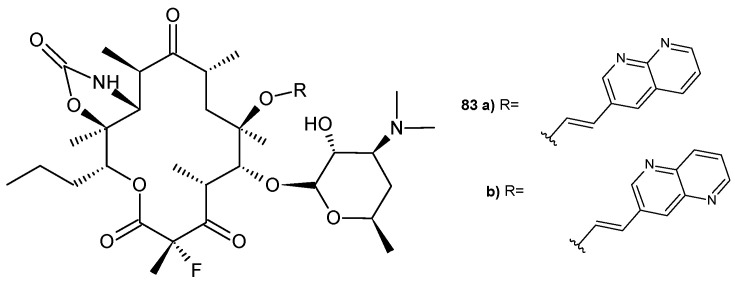
Ketolides containing naphthyridine scaffold, **83a**–**b**.

D. Abbanat et al. [89] prepared a ketolide combined with 1,8-naphthyridine, **83a** (Figure 56). Compound **83a** exhibited good activity (MIC = 0.25 µg/mL) against a macrolide-resistant strain of *S. pneumoniae.*

## 3. 1,5-Naphthyridine Derivatives

### 3.1. 1,5-Naphthyridines Conjugated with Antibiotics

The same researchers [89] also tested the ketolide conjugated with a 1,5-naphthyridine scaffold, **83b** (Figure 56), against the same macrolide-resistant strain of *S. pneumoniae*. Ketolides **83a**–**b** inhibited protein synthesis in vitro. The MIC_90_ values of the tested ketolides were several times lower than those of telithromycin and erythromycin A [89]. 

**Figure 57:**
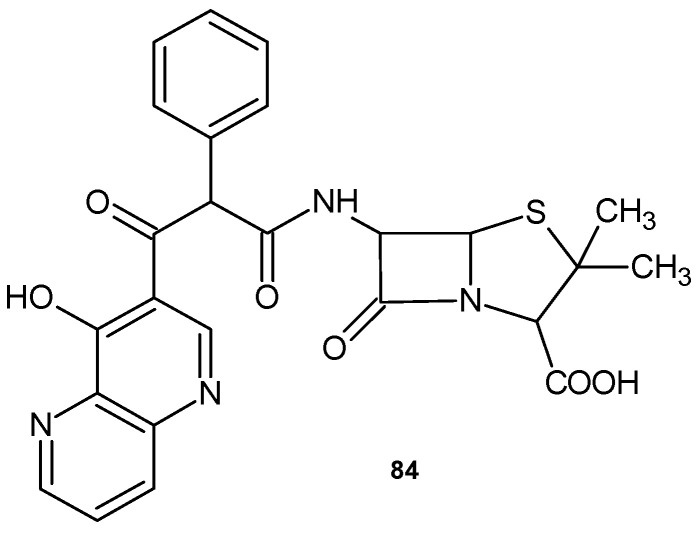
6{D(-)-a(4-hydroxyl-1,5-naphthyridine-3-carboxamido)phenylacetamido} sodium penicillinate **84**.

Modifications of the penicillin molecule have led to progress in antibiotic therapy. 6{D(-)-α(4-hydroxyl-1,5-naphthyridine-3-carboxamido)phenylacetamido} sodium penicillinate, **84** (Figure 57), is a semisynthetic penicillin with a broad spectrum of activity against Gram-positive cocci and Gram-negative bacilli. Its activity against *P. aeruginosa* is similar to that of aminoglycoside antibiotics [90].

### 3.2. Synthetic 1,5-Naphthyridine Derivatives

**Figure 58:**
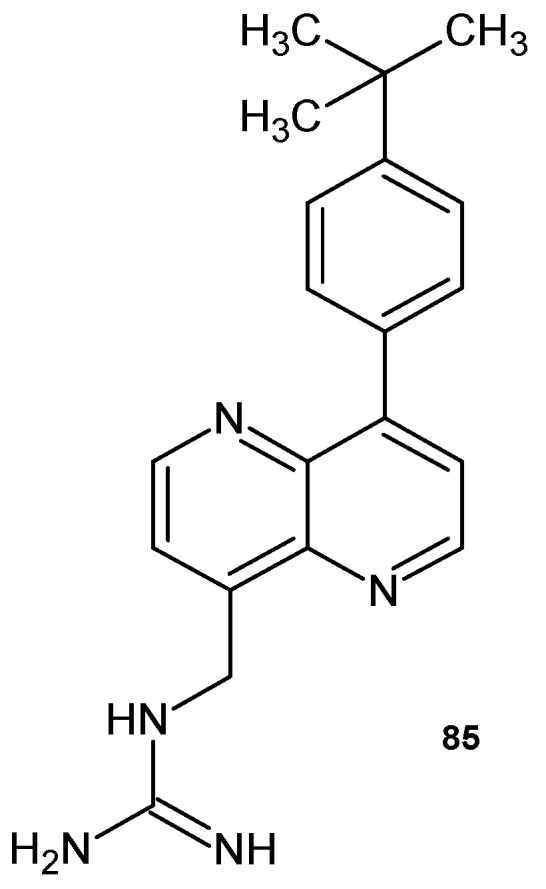
*N*-{[8-(4-*tert*-butylphenyl)-1,5-naphthyridin-4-yl]methyl}guanidine **85**.

A.K. Parhi et al. [91] prepared *N*-{[8-(4-*tert*-butylphenyl)-1,5-naphthyridin-4-yl]methyl}guanidine **85** (Figure 58), which showed antibacterial activity against methicillin-sensitive *S. aureus* (MSSA) and methicillin-resistant *S. aureus* (MRSA), with MIC = 8.0 μg/mL.

**Figure 59:**
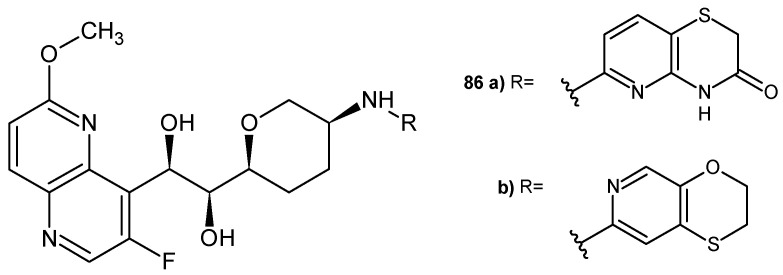
2-(3-Fluoro-6-methoxy-1,5-naphthyridin-4-yl)ethane-1,2-diol derivatives **86a**–**b**.

J-P. Surivet et al. [92] obtained 3-fluoro-6-methoxy-1,5-naphthyridine derivatives as inhibitors of bacterial type II topoisomerases (topoisomerase IV and DNA gyrase). The most potent compounds, **86a**–**b** (Figure 59), were found to be dual inhibitors of DNA gyrase and topoisomerase IV, with broad antibacterial activity and a low spontaneous development of resistance. (1*S*,2*R*)-1-((2*S*,5*R*)-5-(((2,3-dihydro-[1,4]oxathiino [2,3-*c*]pyridin-7-yl)methyl)amino)tetrahydro-2*H*-pyran-2-yl)-2-(3-fluoro-6-methoxy-1,5-naphthyridin-4-yl) ethane-1,2-diol **86b** also showed moderate clearance in rats and satisfactory in vivo efficacy against *S. aureus* in a murine model of infection.

**Figure 60:**
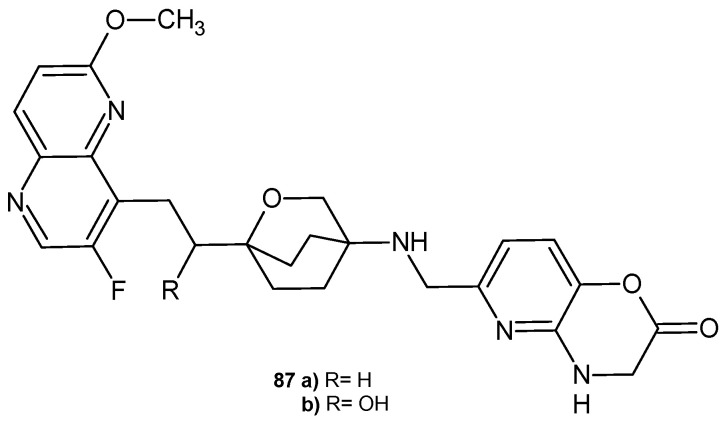
3-Fluoro-6-methoxy-1,5-naphthyridine derivatives **87a**–**b**.

S. B. Singh et al. [93] also synthesized 3-fluoro-6-methoxy-1,5-naphthyridine derivatives containing an oxabicyclooctane linker, but with a pyridoxazinone moiety, **87a**–**b** (Figure 60), as inhibitors of bacterial type II topoisomerases (topoisomerase IV and DNA gyrase). These compounds showed broad-spectrum antibacterial activity against MRSA and Gram-negative pathogens *Acinetobacter baumannii* and *E. coli*. They showed activity against quinolone-resistant *S. aureus* and *S. pneumoniae* strains, but exhibited weaker activity against *P. aeruginosa*. The S enantiomer (α-hydroxy) of **87b** was slightly more potent than its R enantiomer. Both derivatives showed potent activity against *S. aureus* and *E. coli* gyrase and better activity against *E. coli* topoisomerase IV compared to *S. aureus* topoisomerase IV. Compounds **87a**–**b** were stable in human, dog, and mouse liver microsomal incubations.

The 2-hydroxy derivative **87b** showed improved in vivo efficacy in a murine *S. aureus* bacteremia survival model. The influence of other substituents instead of the hydroxyl group on the activity of the derivatives was investigated [94]. Single and double substitutions with OH, NH_2_, COOH, F, and CH_3_ groups indicated that a single hydroxyl substitution at position 2 is preferred for potency. Furthermore, the monohydroxy compound showed better efficacy after intravenous administration in the murine model of *S. aureus* infections. The influence of substituents in the 1,5-naphthyridine system on the activity of the derivatives was also tested [95]. The attachment of methoxy and CN groups at C-2 and halogen and hydroxy at C-7 seems to be acceptable, while substitutions at the remaining three carbons generally have a detrimental effect on the antibacterial activity. Substitution of the fluorine atom with a chlorine or CN group did not result in loss of in vitro activity but only weaker properties in the in vivo *S. aureus* infection model [95].

**Figure 61:**
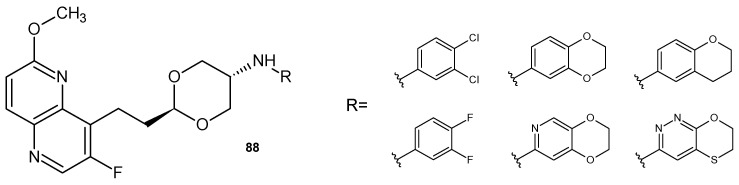
2-[2-(3-Fluoro-6-methoxy-1,5-naphthyridin-4-yl)ethyl]-1,3-dioxan-5-amine derivatives **88**.

L. Li et al. [96] also prepared DNA gyrase and topoisomerase IV inhibitors. A series of dioxane-linked 3-fluoro-6-methoxy-1,5-naphthyridine derivatives **88** (Figure 61), showed improved activity against methicillin-resistant *S. aureus*, penicillin-resistant *S. pneumoniae*, vancomycin-resistant *Enterococcus faecium*, and *S. pyogenes*. These compounds also reduced hERG inhibition. 

**Figure 62:**
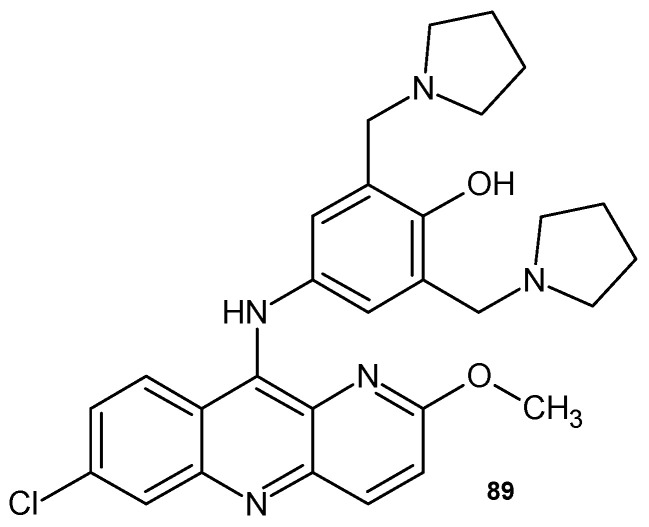
Pyronaridine **89**.

Pyronaridine **89** (Figure 62) is a Mannich base derivative of 1,5-naphthyridine. 2-Methoxy-7-chloro-10-[3,5-bis(pyrrolidinyl-1-methyl-)4-hydroxyphenyl]aminobenzyl-1,5-naphthyridine **89** has been clinically tested as an antimalarial drug effective in treating malaria-infected patients in chloroquine resistance regions [97]. It has been used as an antimalarial agent against *Plasmodium falciparum* and *Plasmodium vivax* since the 1970s. The combination of pyronaridine **89** and artesunate (Pyramax@) has been found to provide more potent antiviral activity with less toxicity and is therefore being investigated as a possible treatment for SARS-CoV-2 [98].

**Figure 63:**
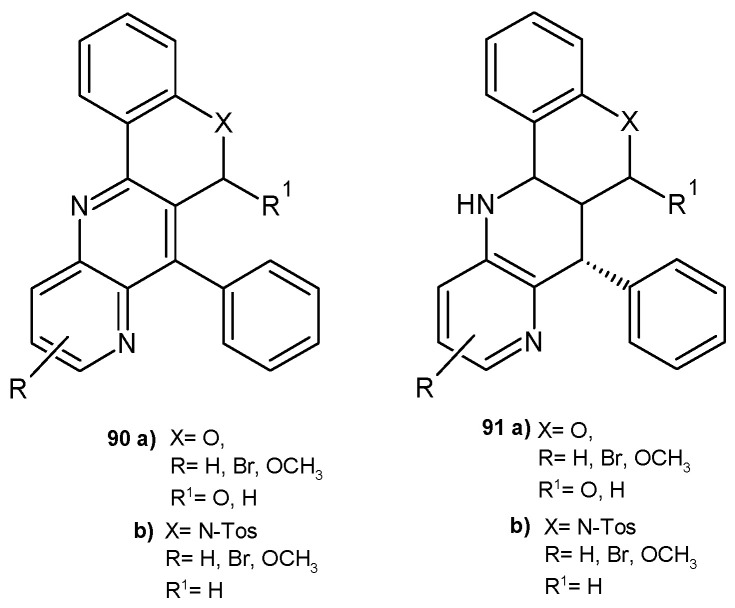
Chromeno [4,3-*b*]naphthyridines and quinolino [4,3-*b*]naphthyridines **90**–**91**.

E. Melcón-Fernandez et al. [86] prepared a series of fused 1,5-naphthyridines, **90**–**91** (Figure 63), and 1,8-naphthyridines, **75**–**78** (Figure 53), and evaluated their antileishmanial activity using in vitro and ex vivo assays. 1,8-Naphthyridines **75**–**78** showed better activity against leishmaniasis than 1,5-naphthyridine derivatives **90**–**91**. The presence of a nitrogen atom in the ring fused to the naphthyridine system was important for the increased activity against *L. infantum* amastigotes. Naphthyridines fused to quinoline were more active than those fused to the chromene ring. Quinoline derivatives exhibited EC_50_ values in the range of 4.93–5.53 µM and were also less toxic.

**Figure 64:**
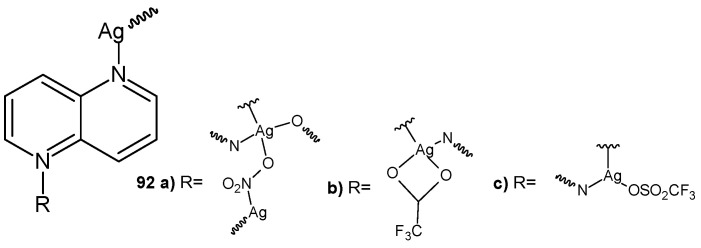
Silver(I) complexes with 1,5-naphthyridines **92a**–**c**.

S. Durić et al. [99] prepared silver(I) complexes with 1,5-naphthyridine: [Ag(NO_3_)(1,5-naph)]n **92a**, [Ag(CF_3_COO)(1,5-naph)]n **92b**, and [Ag(CF_3_SO_3_)(1,5-naph)]n **92c** (Figure 64). The obtained complexes showed good antibacterial activity, with MIC values in the range of 2.5–100 μg/mL, and better antifungal activity against *Candida spp*., with MIC values in the range of 0.78–6.25 μg/mL. Compound **92c** showed the best therapeutic potential due to the lowest MIC values against the tested *Candida* strains, as well as a non-toxic in vivo response in zebrafish embryos. Complexes **92a** and **92b** effectively inhibited *C. albicans* biofilms, while complex **92a** also inhibited the formation of mixed C*. albicans/P. aeruginosa* biofilms [99].

### 3.3. Alkaloids Containing a 1,5-Naphthyridine Scaffold

**Figure 65:**
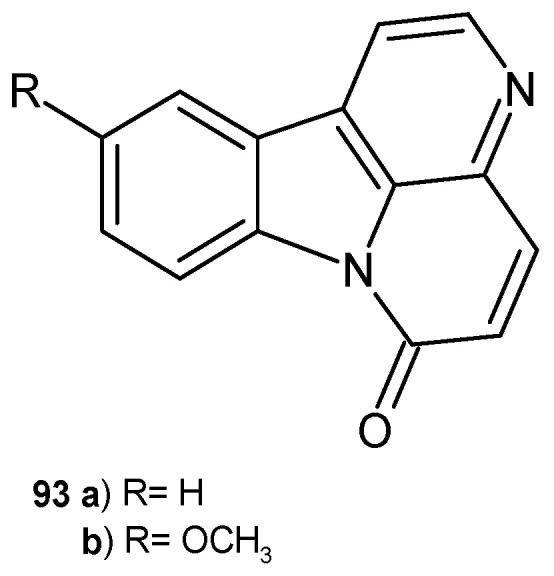
Canthin-6-one **93a** and 10-methoxycanthin-6-one **93b**.

Canthin-6-one **93a** and 10-methoxycanthin-6-one **93b** (Figure 65) were isolated from *Zanthoxylum paracanthum* as promising antimicrobial substances [100]. Alkaloids **93a**–**b** showed strong activity against methicillin-resistant *S. aureus* strains, with MIC values of 0.98 µg/mL and 3.91 µg/mL, respectively. Antifungal activity against *C. albicans* was presented, with MIC values of 3.91 and 7.81 µg/mL for canthin-6-one **93a** and 10-methoxycanthin-6-one **93b**, respectively. Moreover, 10-hydroxycanthin-6-one **93b** showed antifungal effects against *Fusarium graminearum* and *Fusarium solani* (growth inhibition rate 74.5% and 57.9%), and antibacterial activity against *B. cereus*, with MIC = 15.62 µg/mL [101]. Alkaloids **93a**–**b** also showed antimycobacterial effects against *M. smegmatis*, *M. phlei*, and *M. fortuitum*, with MIC values in the range of 2–8 µg/mL [102]. Canthin-6-one **93a** exhibited antiparasitic activity in mice infected with *Trypanosoma cruzi*. Due to its low toxicity, alkaloid **93a** is a promising candidate for the treatment of Chagas disease [103].

## 4. 1,6-Naphthyridine Derivatives

### 4.1. Alkaloids Containing a 1,6-Naphthyridine Scaffold

**Figure 66:**
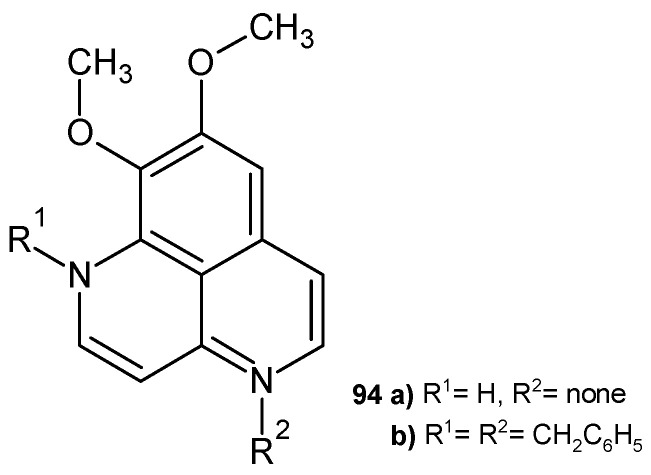
Aaptamine **94a** and 1,4-dibenzylaaptamine **94b**.

Aaptamine (8,9-dimethoxy-1*H*-benzo[*de*][1,6]naphthyridine **94a** (Figure 66) isolated from *Aaptos aaptos* displayed antiviral activity against HIV-1 [104]. Alkaloid **94a** and its dibenzyl derivative **94b** showed anti-amoebic effect towards *Acanthamoeba castellanii*, with IC_50_ = 45 µg/mL and IC_50_ = 8 µg/mL, respectively [105]. 

### 4.2. Synthetic 1,6-Naphthyridine Derivatives

**Figure 67:**
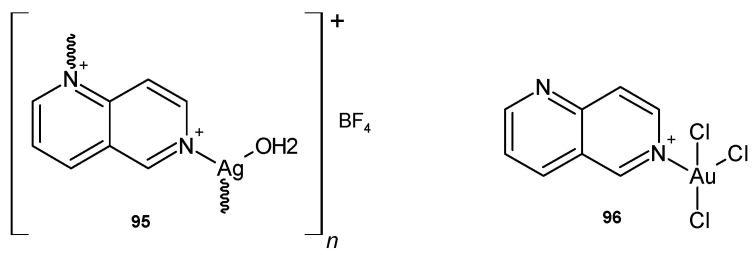
Silver(I) and gold(III) coordination compounds with 1,6-naphthyridine scaffold.

D.P. Aśanin et al. [106] prepared silver(I) and gold(III) coordination compounds with 1,6-naphthyridine scaffolds. {[Ag(1,6-naph)(H_2_O)](BF_4_)}_n_ **95** (Figure 67) showed good antifungal activity against *C. albicans* and *Candida parapsilosis*, with MIC values of 0.49 and 3.9 µg/mL, respectively, while no significant antibacterial activity was observed against *S. aureus*, *L. monocytogenes*, *P. aeruginosa*, *E. coli*, and *K. pneumoniae*. [AuCl_3_(1,6-naph)] **96** (Figure 67) exhibited moderate activity only against *S. aureus*, *L. monocytogenes*, *P. aeruginosa*, and *E. coli*, with MIC = 62.5 µg/mL. Both polymers **95**–**96** were less cytotoxic to human health fibroblast cell lines than the silver-based antimicrobial drug. 

**Figure 68:**
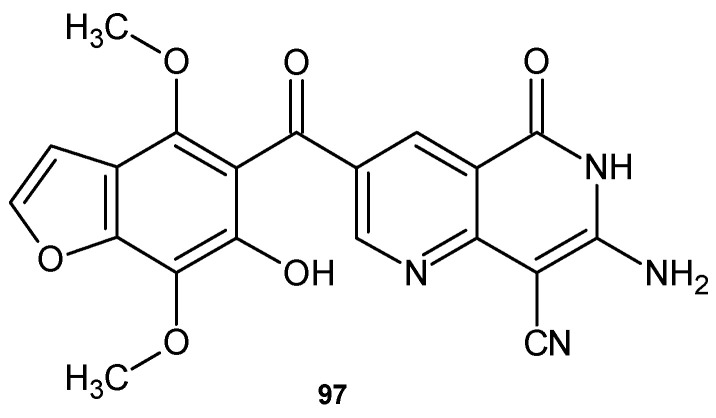
7-Amino-3-[(6-hydroxy-4,7-dimethoxy-1-benzofuran-5-yl)carbonyl]-5-oxo-5,6-dihydro-1,6-naphthyridine-8-carbonitrile **97**.

Ibrahim and El-Gohary [107] obtained 7-amino-3-[(6-hydroxy-4,7-dimethoxy-1-benzofuran-5-yl)carbonyl]-5-oxo-5,6-dihydro-1,6-naphthyridine-8-carbonitrile **97** (Figure 68), with high antibacterial activity against Gram-positive strains *S. aureus* and *B. subtilis* and moderate activity against Gram-negative bacteria strains *E. coli* and *S. typhimurium*. This compound **97** also showed moderate antifungal activity against *C. albicans* and *A. fumigatus*.

**Figure 69:**
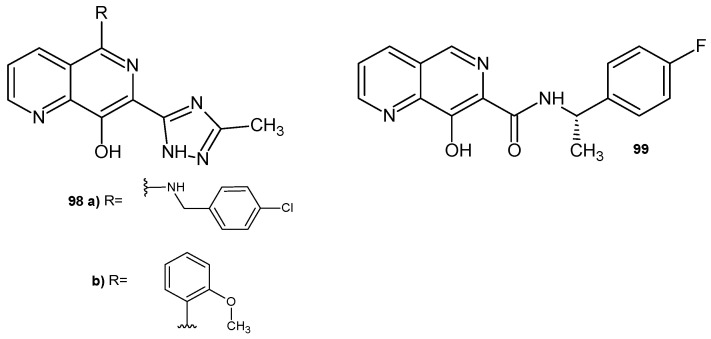
8-Hydroxy-1,6-naphthyridine derivatives **98**–**99**.

8-Hydroxy-1,6-naphthyridine derivatives **98**–**99** (Figure 69) were synthesized by R. J. Wall et al. [108] and showed high activity against *Trypanosoma brucei* and *Leishmania donovani*. The influence of divalent cations Ca^2+^, Cu^2+^, Fe^2+^, Mg^2+^, and Mn^2+^ on the potency of derivatives **98**–**99** against *T. brucei* was also assessed. The addition of FeCl_2_ led to a significant increase in the EC_50_ of the tested compounds. In contrast, the addition of tolerated levels of other divalent cations had little effect on the potency of these compounds. The addition of 100 M FeCl_2_ reduced the potency of the tested compounds in the case of *L. donovani.*

## 5. 2,6-Naphthyridine Derivatives

### 5.1. Synthetic 2,6-Naphthyridine Derivatives

**Figure 70:**
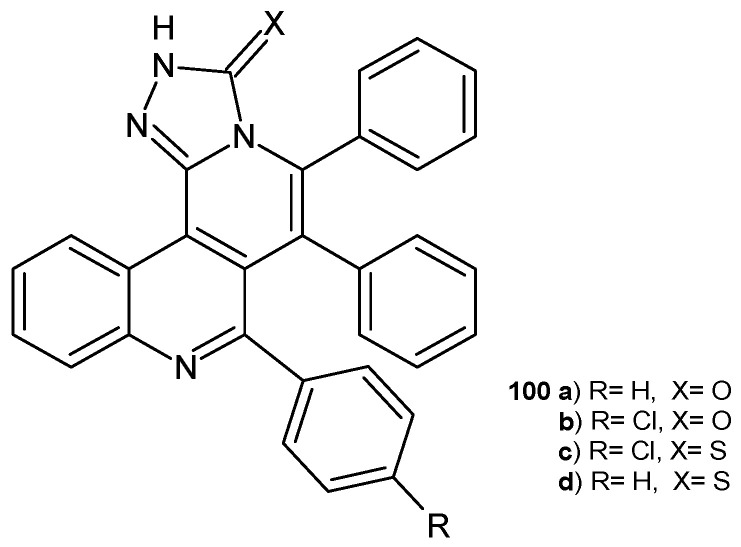
Benzo[*h*][1,2,4]triazolo[3,4-*a*][2,6]naphthyridine derivatives **100a**–**d**.

A. Bishnoi et al. [109] synthesized benzo[*h*][1,2,4]triazolo [3,4-*a*][2,6]naphthyridine derivatives as potential antibacterial agents. The activity of the obtained compounds, **100a**–**d** (Figure 70), was tested against Gram-positive and Gram-negative bacterial strains *S. aureus*, *B. subtilis*, *P. aeruginosa*, and *E. coli*, as well as on fungal strains *C. albicans*, *A. niger*, and *A. fumigatus* using the disc diffusion method. No activity was observed against *A*. *niger.* Compounds **100b**–**c** exhibited good activity against *C. albicans*, with MIC values of 12.5 mg/mL and 25 mg/mL, respectively. Compound **100a** showed activity against *A. fumigatus*, with MIC = 25 mg/mL. Compounds **100d** and **100c** were the most active antibacterial agents, with MIC values of 12.5 mg/mL against *B. subtilis* and *S. aureus* [109].

### 5.2. An Alkaloid Containing a 2,6-Naphthyridine Scaffold

**Figure 71:**
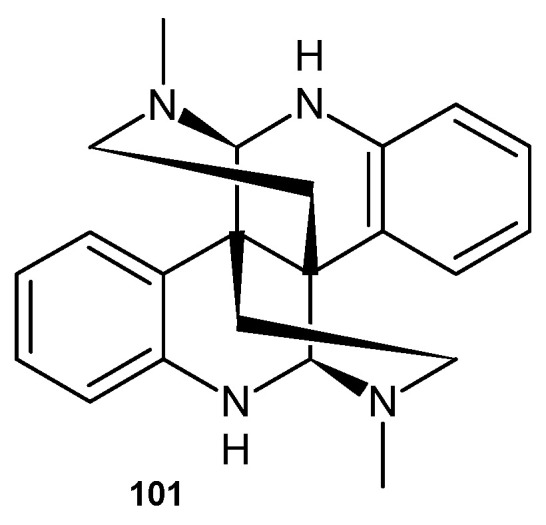
Calycanthine **101**.

Calycanthine **101** (Figure 71)—an alkaloid containing a 2,6-naphthyridine scaffold, which was isolated from *Chimonanthus praecox* seeds—exhibited significant activity against two plant pathogenic fungi, *Exserohilum turcicum* and *Bipolaris maydis*, with EC_50_ values of 103.1 and 29.3 mg/mL, respectively [110].

## 6. 2,7-Naphthyridine Derivatives

### 6.1. Synthetic 2,7-Naphthyridine Derivatives

**Figure 72:**
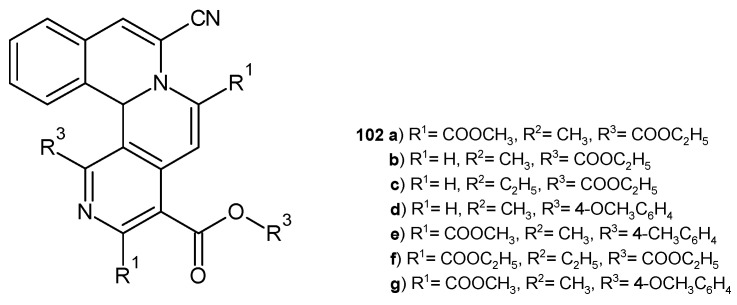
Isoquino[1,2-*a*][2,7]naphthyridine derivatives **102a**–**g**.

Z. Hossaini et al. [111] investigated the antibacterial activity of synthesized isoquino [1,2-*a*][2,7]-naphthyridine derivatives **102a**–**g** (Figure 72). The evaluation displayed that compounds **102a**–**d** exhibit very good activity against *S. aureus*, *B. cereus*, *E. coli*, and *K. pneumoniae* strains, comparable to streptomycin and gentamicin. 

**Figure 73:**
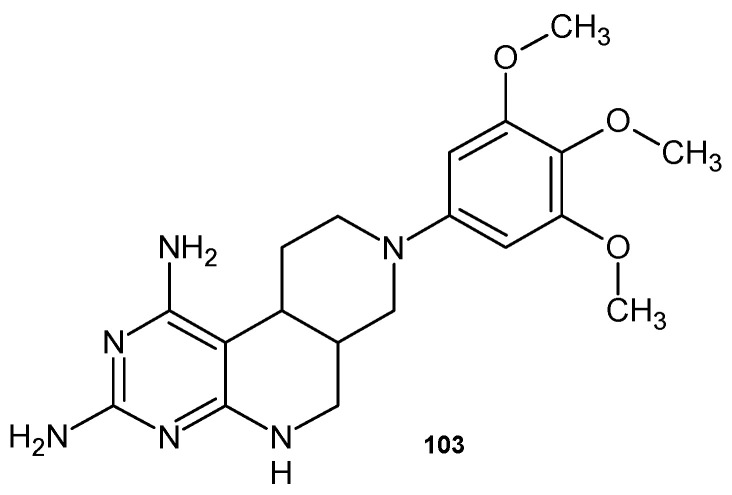
8-(3,4,5-Trimethoxyphenyl)-5,6,6a,7,8,9,10,10a–octahydropyrimido[4,5-*c*][2,7]naphthyridine -1,3-diamine **103**.

Pyrimido[4,5-*c*][2,7]naphthyridine derivatives obtained by A. Gangjee et al. [112] were evaluated as inhibitors of dihydrofolate reductase (DHFR) from *Pneumocystis carinii* and *Toxoplasma gondii*. The most potent analogue was the trimethoxyphenyl derivative **103** (Figure 73), with IC_50_ = 86 nM. 

**Figure 74:**
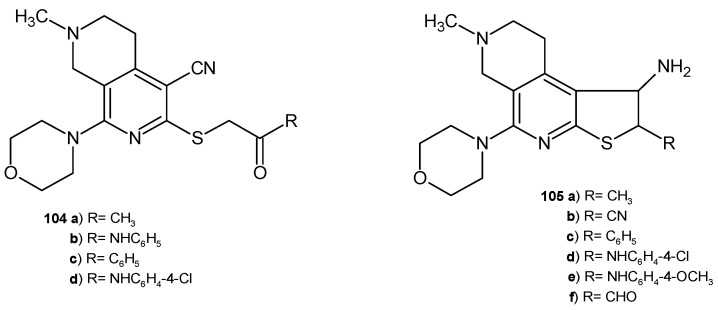
2,7-Naphthyridine-4-carbonitrile derivatives **104** and thieno [2,3-*c*][2,7]naphthyridine derivatives **105**.

2,7-Naphthyridine-4-carbonitrile derivatives **104** and thieno[2,3-*c*][2,7]naphthyridine derivatives **105** (Figure 74) were evaluated for their antimicrobial activity against four bacterial strains (*E. coli*, *S. aureus*, *P*. *aeruginosa*, and *B. cereus*) and four fungal strains (*Geotrichum candidium*, *C. albicans*, *Trichophyton rubrum*, and *A. flavus*) by measuring the inhibition zones [113]. The acetyl group in this structure enhanced the strong antibacterial effect. Compounds **104a**–**b** and **105a**,**d** were the most potent against *B. cereus* and *E. coli*, with MIC values in the range of 7.0–8.0 μg/mL. Derivatives **105a,b,e** showed significant activity against *S. aureus*, with MIC values in the range of 6.0–8.0 μg/mL. The *P. aeruginosa* strain turned out to be sensitive only to compound **104a** (MIC = 6.0 μg/mL). Substitution of the acetyl group with a benzoyl group (compound **105c**) resulted in strong antifungal activity against *G. candidium*, *T. rubrum*, and *A. flavus*, with MIC values in the range of 7.0–9.0 μg/mL. Derivatives **105c** and **105f** showed very good antifungal activity against *A*. *flavus* and *T. rubrum*, with MIC values in the range of 7.0–8.0 μg/mL. *C. albicans* was sensitive to the 4-chlorophenyl derivatives **104d** and **105d** (MIC= 8.0 μg/mL) [113].

### 6.2. Alkaloids Containing a 2,7-Naphthyridine Scaffold

**Figure 75:**
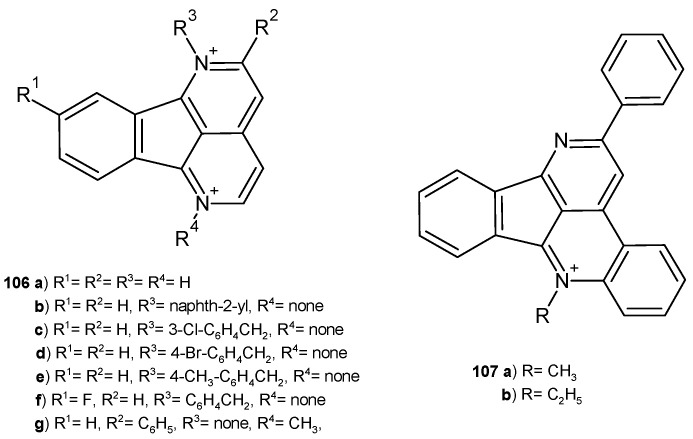
Eupolauridine and its derivatives, **106**–**107**.

Eupolauridine-indeno[1,2,3-*ij*][2,7]naphthyridine **106a** (Figure 75), extracted from the root bark of *Cleistopholis patens* by Hufford et al. [114], exhibited in vitro efficacy against fungal pathogens *C. albicans*, *Cryptococcus neoformans*, and *A. fumigatus* and selective inhibition of fungal topoisomerase I [115]. This alkaloid **106a** showed significant activity against *C. albicans*, with MIC = 5.25 μg/mL [115]. S. Taghavi-Moghadam et al. [116] prepared eupolauridine derivatives **106**–**107** (Figure 75), which were evaluated for their antimicrobial activity. Compounds **106b**, **106g**, and **107a**–**b** exhibited antifungal activity toward *C. albicans*, with MIC values in the range of 0.098–0.78 µg/mL, and compounds **106b**–**g** and **107a**–**b** acted similarly toward *C. neoformans*, with MIC values in the range of 0.39–0.78 µg/mL. However, none of the synthesized analogues **106**–**107** were effective against *A. fumigatus.* Only compounds **107a**–**b** showed very good antibacterial activity against *S. aureus*, *MRS*, and *P. aeruginosa*, with IC_50_ values in the range of 2–5 µg/mL. Derivatives **106c** and **106e** showed significant antimalarial activity. 

**Figure 76:**
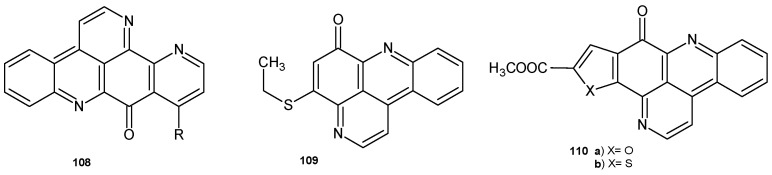
Pyrido[2,3,4-*kl*]acridin-6-one derivatives **108**–**110**.

Ascididemin **108** (Figure 76) exhibited antimicrobial activity against *Cladosporium resinae*, *E. coli*, and *B. subtilis* and also very good potency (MIC = 0.35 mM) against *Mycobacterium tuberculosis* [117]. Pyrido[2,3,4-*kl*]acridin-6-one derivatives prepared by D.R. Appleton et al. [118] were evaluated for their ability to inhibit the growth of *M. tuberculosis* in vitro. The most potent were 4-ethylthiopyrido[2,3,4-*kl*]acridin-6-one **109**, with an MIC = 0.34 mM, and **110a**–**b**, with MIC values of 0.58.5 mM and 0.61 mM, respectively.

**Figure 77:**
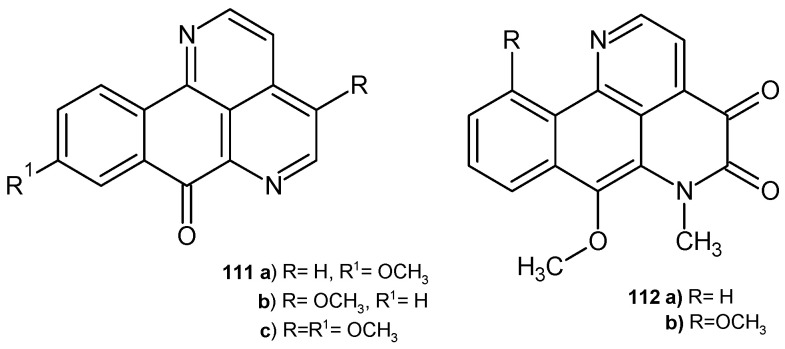
Eupomatidines **111a**–**c,** imbiline **112a,** and hadranthine A **112b**.

Kitahara et al. [119] obtained eupomatidines **111a**–**c** (Figure 77), which were evaluated for their antifungal activity against *C. albicans*, *Paecilomyces variotii*, and *Trichophyton mentagrophytes*. Eupomatidine-1 **111a** showed activity against all strains, with EC_50_ values of 50 μg/mL, 6.25 μg/mL, and 0.4 μg/mL, respectively. Eupomatidines **111b**–**c** were active only toward *T. mentagrophytes*, with EC_50_ values of 3.1 μg/mL and 6.25 μg/mL, respectively. 

7-Methoxy-6-methyl-4,5-dihydronaphthol[1,2,3-*ij*][2,7]naphthyridine-4,5-(6*H*)-dione derivatives imbiline **112a** and hadranthine A **112b** (Figure 77), isolated from *Duguetia hadrantha*, were evaluated for their antimalarial and antimicrobial activities. These alkaloids showed weak antimalarial potency against chloroquine-resistant *Plasmodium falciparum*. Imbiline **112a** was found to be inactive against *S. aureus*, as well as against fungal strains *C. albicans* and *C. neoformans*. Hadranthine A **112b** exhibited activity against *C. albicans*, with MIC = 20 μg/mL [120]. 

**Figure 78:**
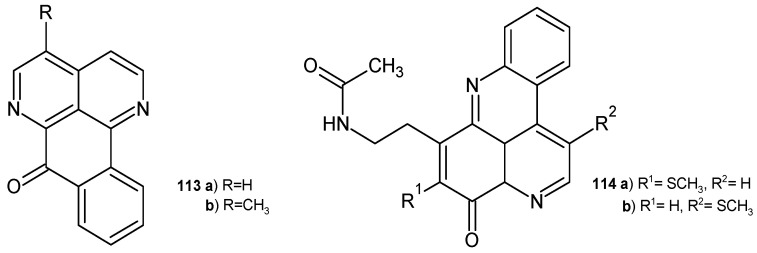
Sampangines **113a**–**b** and diplamines **114a**–**b**.

Sampangine **113a** and 3-methoxysampangine **113b** (Figure 78) were also isolated from *D. hadrantha* and then evaluated for their antimalarial, antifungal, and cytotoxic potential [121]. Both alkaloids **113a**–**b** showed activity against *P. falciparum* without cytotoxicity toward VERO cells. These alkaloids **113a**–**b** also exhibited activity against *Mycobacterium intracellulare*, comparable to rifampin [122]. Sampangine **113a** was also found to be a potent antifungal agent against *Paecilomyces variotii* and *Trichophyton mentagrophytes*, with EC_50_ = 0.2 μg/mL [120]. 3-Methoxysampangine **113b** showed significant antifungal activity against *C. albicans*, *C. neoformans*, and *A. fumigatus*, better than amphotericin B [122]. 

Diplamines **114a**–**b** (Figure 78), isolated from the tunicate *Diplosoma* sp. [123], exhibited moderate antimicrobial activity towards *B. subtilis*, *E. coli*, *C. albicans*, and *T. mentagrophytes* [124]. 

**Figure 79:**
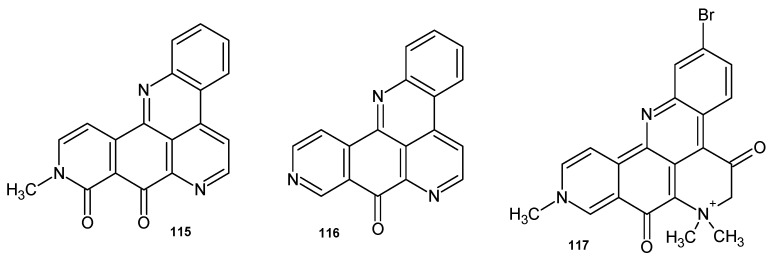
Amphimedine analogues **115–117**.

Amphimedine analogues **115–117** (Figure 79) isolated from marine sponges were evaluated for their antimicrobial activities. Neoamphimedine **116** showed antitrypanosomal activity against *T. brucei*, with IC_50_ = 0.21 μM. Demethyldeoxyamphimedine **116** exhibited antibacterial activity against *Listonella anguillarum* and *M. luteus* [125]. Petrosamine B **117** inhibited *Helicobacter pylori* aspartyl semialdehyde dehydrogenase (IC_50_ = 306 μM) [126]. 

**Figure 80:**
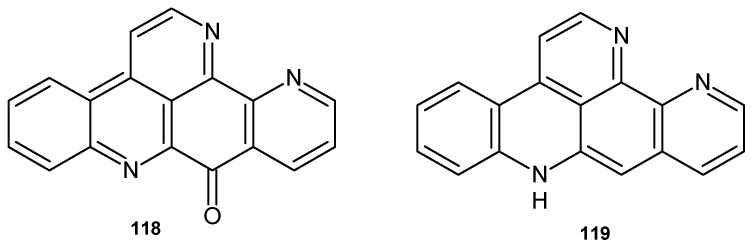
Ascididemines **118**–**119**.

Ascididemin **118** and 12-deoxyascididemin **119** (Figure 80) showed significant activity against *T. brucei*, with IC_50_ values of 0.077 and 0.032 μM, respectively [127]. Ascididemin **118** exhibited antimicrobial activity against *C. resinae*, *E. coli*, and *B. subtilis* [117] and very good efficacy against *M. tuberculosis*, with MIC = 0.35 μM [118].

## 7. Conclusions

Naphthyridine derivatives have been studied for their various pharmacological activities. This review presents the literature data on synthetic and natural naphthyridine derivatives that have been reported to possess antimicrobial activity. Most publications refer to the 1,8-naphthyridine isomer, since the first naphthyridine derivative introduced into therapy in 1967 was the antibacterial nalidixic acid. Since then, several other 1,8-naphthyridine derivatives (mainly fluoro derivatives) have been approved for therapy, as well as one derivative of the 1,5-naphthyridine isomer as the antimalarial drug pyronaridine. Although the vast majority of naphthyridines with antimicrobial properties are derivatives of the 1,8-isomer, many scientific reports also refer to the 1,5-naphthyridine derivatives. So far, no 1,7-naphthyridine derivative has been obtained that would exhibit antimicrobial activity. Alkaloids containing in their structure the 1,5-, 1,6-, 2,6- or 2,7-naphthyridine system have also been isolated [128]. The antibacterial, antifungal, antimalarial, or antiparasitic properties of these alkaloids have been studied. Some of them were also active against drug-resistant strains. Canthin-6-one, 10-methoxycanthin-6-one, and eupolauridine derivatives were active against methicillin-resistant *S. aureus*, while imbiline and hadranthine A were active against chloroquine-resistant *P. falciparum*. Despite the broad antimicrobial activity of naphthyridine derivatives, the molecular mechanisms of their action are still poorly understood, which makes them an interesting target for deeper research. We believe that this review will contribute to further research on the properties of naphthyridine derivatives, which will allow the use of these compounds in the fight against the increasing drug resistance of microorganisms. 

## Data Availability

No new data were created or analyzed in this study. Data sharing is not applicable to this article.
